# An Integrated INS/LiDAR SLAM Navigation System for GNSS-Challenging Environments

**DOI:** 10.3390/s22124327

**Published:** 2022-06-07

**Authors:** Nader Abdelaziz, Ahmed El-Rabbany

**Affiliations:** 1 Department of Civil Engineering, Ryerson University, Toronto, ON M5B 2K3, Canada; rabbany@ryerson.ca; 2 Department of Civil Engineering, Tanta University, Tanta 31527, Egypt

**Keywords:** Kitware SLAM, INS/LiDAR SLAM integration, integrated navigation system

## Abstract

Traditional navigation systems rely on GNSS/inertial navigation system (INS) integration, in which the INS can provide reliable positioning during short GNSS outages. However, if the GNSS outage persists for prolonged periods of time, the performance of the system will be solely dependent on the INS, which can lead to a significant drift over time. As a result, the need to integrate additional onboard sensors is essential. This study proposes a robust loosely coupled (LC) integration between the INS and LiDAR simultaneous mapping and localization (SLAM) using an extended Kalman filter (EKF). The proposed integrated navigation system was tested for three different driving scenarios and environments using the raw KITTI dataset. The first scenario used the KITTI residential datasets, totaling 48 min, while the second case study considered the KITTI highway datasets, totaling 7 min. For both case studies, a complete absence of the GNSS signal was assumed for the whole trajectory of the vehicle in all drives. In contrast, the third case study considered the use of minimal assistance from GNSS, which mimics the intermittent receipt and loss of GNSS signals for different driving environments. The positioning results of the proposed INS/LiDAR SLAM integrated system outperformed the performance of the INS for the residential datasets with an average reduction in the root mean square error (RMSE) in the horizontal and up directions of 88% and 32%, respectively. For the highway datasets, the RMSE reductions were 70% and 0.2% for the horizontal and up directions, respectively.

## 1. Introduction

Providing accurate, robust positioning is a major challenge that exists in the field of vehicular navigation research and development. Vehicular positioning demands navigation systems that provide an accurate position for all driving environments (i.e., urban or rural), all weather conditions (i.e., dry, rain, snow), and different illumination conditions (i.e., day or night). In addition, the navigation system must possess redundancy, such that the system will still be functional should one sensor fail to operate. As a result, the use of a single sensor may not produce a robust navigation solution, even if such a sensor produces measurements that lead to accurate positioning. This emphasizes the need for multi-sensor integration to achieve robust positioning [1,2].

GNSS/INS integrated navigation systems are the most popular. Typically, the observations of the GNSS and IMU are fused using a Kalman filter [3]. For example, in a loosely coupled (LC) integration, the INS provides the position of the vehicle through mechanization, while receiving updates from the GNSS at a slower frequency to minimize the INS solution drift. The integration relies on the ability of the INS to provide the position of the vehicle at a high frequency. As a result, for closed-error scheme integration, the INS can provide reliable positioning while experiencing short GNSS signal outages [4]. However, if the GNSS outage occurs for a prolonged period of time, the system will rely on the performance of the INS, which is prone to a significant drift, especially when a low-cost micro-electro-mechanical system (MEMS) inertial measurement unit (IMU) is used [5,6,7,8,9].

In order to improve the performance of navigation systems, additional onboard sensors that can be used for navigation are required which will allow the system to sustain prolonged GNSS outages. LiDAR sensors are widely used for localization through simultaneous mapping and localization (SLAM) techniques. The basic idea of SLAM algorithms is to use a LiDAR or a camera sensor to construct a map of the surrounding environment while simultaneously keeping track of the sensor location [10].

There are several LiDAR SLAM algorithms. In 2014, the LiDAR odometry and mapping (LOAM) algorithm was presented as one of the leading algorithms [11]. This was supported by its superior performance via the KITTI benchmark [12]. Subsequently, other variations and updated versions of LOAM were presented, including, A-LOAM [13], LeGO-LOAM [14], Kitware SLAM [15], R-LOAM [16], and F-LOAM [17]. The common contribution of these SLAM algorithms was improvement in the processing time of the LOAM algorithm. For vehicular navigation, the integration of LiDAR SLAM with GNSS/INS is crucial to achieve system redundancy and robustness.

Many studies have proposed the integration of GNSS, INS, and LiDAR SLAM. In [18], an integration scheme was proposed which integrates GNSS/INS with LiDAR SLAM based on graph optimization. In this study, the GNSS/INS results were matched with the relative pose of a 3D probability map. The system was tested during a one-minute outage of the GNSS signal. The RMSE of the position in the east and north directions was reduced by roughly 80% compared to the GNSS/INS navigation solution. Similar work was undertaken in [19]; however, an odometer was included in the integration scheme. The system was tested during a 2-min outage, which resulted in reductions in the RMSE by 62.8%, 72.3%, and 52.1%, along the north, east, and height directions, respectively, compared to the GNSS/INS integrated system. In [20], the integration of a three-dimensional reduced-inertial-sensor system (3D-RISS) with GNSS and LiDAR odometry was accomplished using an extended Kalman filter (EKF). The LiDAR odometry updates to the 3D-RISS mechanization were the position and azimuth. The system was tested for different driving scenarios in downtown Toronto and Kingston, Canada. The integration results showed a reduction in positioning errors by 64% compared to the inertial navigation solution.

In this paper, an LC integration between the INS and the LiDAR SLAM algorithm using an EKF is proposed. The resulting integrated navigation system produces reliable position and attitude information during complete GNSS signal outages. The main objectives of this paper are as follows: to design an integrated INS/LiDAR SLAM navigation system that satisfies the concept of vehicular navigation robustness and redundancy, where both the LiDAR and the INS sensors provide the position and attitude of the vehicle at moderate and high frequency, respectively; to present an LC integration that takes advantage of the LiDAR SLAM solution, including the 3D position and attitude angles, to update the INS solution in a closed-error loop scheme using the EKF; to test the proposed integrated navigation system on the raw KITTI dataset for both residential driving environments of scenes containing dense features and low driving speed; to test the navigation system in highway driving environments of scenes featuring sparse features and high driving speed; and to simulate and study the effect of intermittent receipt and loss of GNSS signals while driving in various environments.

The remainder of this paper is organized as follows: Section 2 includes the system architecture and mathematical models for the IMU mechanization, LiDAR SLAM, and the EKF; Section 3 illustrates the datasets considered in this paper, along with defining the different case studies; Section 4 includes the results of the analysis; Section 5 presents a comparison between the proposed integrated INS/LiDAR system and three state-of-the-art LiDAR SLAM algorithms; and Section 6 ends the paper with some concluding remarks.

## 2. System Architecture and Mathematical Models

### 2.1. Full IMU Mechanization

The IMU observations are measured with respect to the body frame (b-frame), whose axes are defined as follows: the *Y*-axis is the forward direction, the *X*-axis is the transverse direction, and the *Z*-axis is the up direction, such that the coordinate system is right-handed. In addition, the IMU measurements are referenced to the navigation frame, alias the local-level frame (LLF), which is a right-handed system composed of the east, north, and up axes. Given the raw measurements of the accelerometer and gyroscopes of the IMU into the mechanization, the outputs are position (latitude, longitude, and altitude), velocity (east, north, and up directions), and attitude (roll, pitch, and yaw angles) [4].

### 2.2. LiDAR SLAM

The SLAM algorithm used in this study was the Kitware SLAM [15], which is based on the LOAM algorithm [11]. Kitware SLAM was adopted over GraphSLAM algorithms, as the latter are generally more computationally expensive, such that they consider all pose estimates in the calculation process. By contrast, EKF-based SLAM methods consider the last pose only [21].

The LOAM algorithm is composed of three main stages, namely, point cloud registration, LiDAR odometry, and LiDAR mapping. In the first stage, the point cloud registration is accomplished by extracting and matching feature points among every consecutive pair of point clouds. For every scan, feature points corresponding to edges and planes are extracted based on the smoothness of the local surface. This is described by the c coefficient as presented in Equation (1). Low and high c values correspond to planes and edges, respectively.

(1) $$c=\frac{1}{\left|S|.||X_{\left(k,i\right)}^{L}|\right|}||\sum_{j\in S,j\neq i}\left(X_{\left(k,i\right)}^{L}-X_{\left(k,j\right)}^{L}\right)||$$

where *S* is a set of consecutive points returned by the LiDAR in the same scan, $X_{\left(k,i\right)}^{L}$ are the coordinates of point *i* in sweep *k*, expressed in the LiDAR frame, and $X_{\left(k,j\right)}^{L}$ are the coordinates of point *j* in sweep *k*, expressed in the LiDAR frame.

In order to ensure an even distribution of feature points across the point cloud, the scan is divided into subregions. Moreover, a feature point cannot belong to a surface patch that is parallel to a laser beam or on a boundary on an occluded region. Subsequently, the extracted points of a current scan are projected to the beginning of the consecutive scan, where feature correspondence is executed using the nearest neighbor search. Then, the LiDAR motion is recovered by minimizing the overall point to line *d_ε_* and point to plane $d_{H}$ distances as shown by Equations (2) and (3), respectively.

(2) $$d_{\varepsilon }=\frac{\left|({\tilde{X}}_{\left(k+1,i\right)}^{L}-{\bar{X}}_{\left(k,j\right)}^{L})\times ({\tilde{X}}_{\left(k+1,i\right)}^{L}-{\bar{X}}_{\left(k,l\right)}^{L})\right|}{\left|{\bar{X}}_{\left(k,j\right)}^{L}-{\bar{X}}_{\left(k,l\right)}^{L}\right|}$$

(3) $$d_{H}=\frac{\left|\begin{array}{c} \left({\tilde{X}}_{\left(k+1,i\right)}^{L}-{\bar{X}}_{\left(k,j\right)}^{L}\right)\\ \left({\bar{X}}_{\left(k,j\right)}^{L}-{\bar{X}}_{\left(k,l\right)}^{L})\times ({\bar{X}}_{\left(k,j\right)}^{L}-{\bar{X}}_{\left(k,m\right)}^{L}\right) \end{array}\right|}{\left|({\bar{X}}_{\left(k,j\right)}^{L}-{\bar{X}}_{\left(k,l\right)}^{L})\times ({\bar{X}}_{\left(k,j\right)}^{L}-{\bar{X}}_{\left(k,m\right)}^{L})\right|}$$

where ${\tilde{X}}_{\left(k+1,i\right)}^{L}$, ${\bar{X}}_{\left(k,j\right)}^{L}$, ${\bar{X}}_{\left(k,l\right)}^{L}$, ${\bar{X}}_{\left(k,m\right)}^{L}$ are the coordinates of points *i*, *j*, *l*, and *m* in the LiDAR frame, respectively. In order to account for the point cloud motion distortion, the motion of the LiDAR is assumed to have constant and linear angular velocities during each scan. This allows for the linear interpolation of the pose transform for LiDAR points that are acquired at different times. The result of the scan matching is the transformation between a consecutive pair of point clouds, which is the input to the odometry stage. Thereafter, the transformation is projected into the frame of the first point cloud. Then, the distortion-free point cloud is used to build a map at a frequency of 1 Hz. The map is then used to further refine the pose of the LiDAR by performing the same scan-matching technique between the scan and the map.

While Kitware SLAM uses the base architecture of LOAM, there are some improvements. Firstly, the running time is reduced due to the use of C++ libraries and tools designed for better computational performance. In addition, the algorithm is independent from the ROS and does not rely on hard-coded parameters. It can also run in Windows and Linux operating systems using LidarView software. Furthermore, it is more generalized, such that it runs on several LiDAR sensors, not just the Velodyne. Finally, the algorithm can process point clouds from multiple LiDAR sensors. However, it is important to note that, at present, Kitware SLAM neither performs loop closure, nor does it integrate the IMU measurements in the scan matching of subsequent frames.

**Figure 1:**
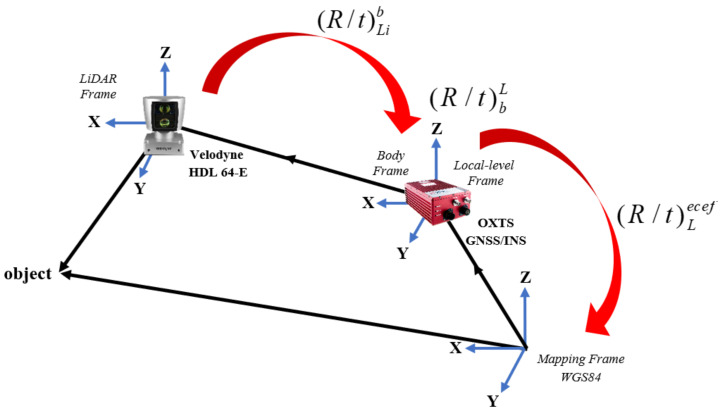
Graphical illustration of LiDAR SLAM pose transformation into the WGS84 reference frame.

After obtaining the SLAM solution in the local frame of the first point cloud of the LiDAR data, it is used to update the inertial navigation solution. However, since the latter is estimated in the WGS84 global reference, the poses estimated from the LiDAR slam must be transformed into the same reference frame, as graphically illustrated in Figure 1.

The position and rotation transformations can be performed using a homogenous transformation using 4 × 4 transformation matrices, which is more computationally efficient. Let *P^Li^* denote a point captured in the local frame of the LiDAR and let denote the same point expressed in the WGS84 reference frame. The sequence of homogenous transformations is presented by Equations (4) and (5).

(4) $$P^{ecef}={\left(R/t\right)}_{l}^{ecef}{\left(R/t\right)}_{b}^{l}{\left(R/t\right)}_{Li}^{b}P^{Li}$$

(5) $$R_{Li}^{l}=R_{b}^{l}R_{Li}^{b}$$

where ${\left(R/t\right)}_{l}^{ecef}$ is the homogenous transformation matrix from the *l*-frame to the WGS84 frame, ${\left(R/t\right)}_{b}^{l}$ is the homogenous transformation matrix from the *b*-frame to the *l*-frame, ${\left(R/t\right)}_{Li}^{b}$ is the homogenous transformation matrix from the LiDAR frame to the *b*-frame, $R_{Li}^{l}$ is the rotation matrix from the LiDAR frame to the *l*-frame, and $R_{Li}^{b}$ is the rotation matrix from the LiDAR frame to the *b*-frame.

### 2.3. INS/LiDAR Integration

**Figure 2:**
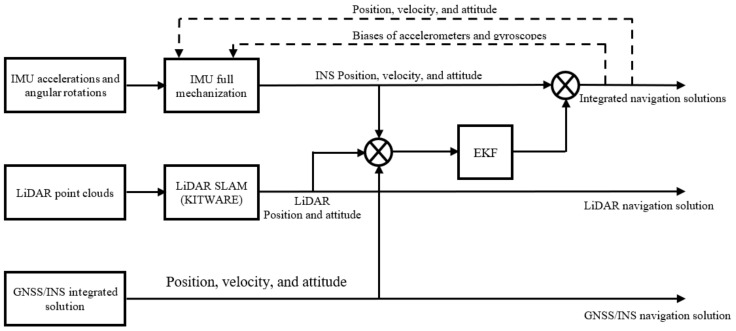
A block diagram for the INS/LiDAR SLAM LC integration.

In this paper, an LC integration between the INS and the LiDAR SLAM is adopted using an EKF, which results in an integrated navigation solution, as shown in Figure 2. The raw IMU measurements of accelerations and angular rotations are used as input to a full IMU mechanization, which outputs the position, velocity, and attitude angles of the navigation system. Meanwhile, the LiDAR point clouds are fed into the Kitware SLAM algorithm, which yields the position and attitude angles of the vehicle. These are used as the measurement update to the IMU mechanization output during the update stage of the EKF, which yields an integrated navigation solution; the updated errors are fed back into the IMU mechanization, which forms a closed-loop error scheme.

### 2.4. System Model

The discrete form of the system model [4] is described by Equations (6) and (7), which are used to predict the error state of the system. The uncertainty of the predicted system error state is estimated by the a priori covariance matrix *P_k_* (−), as presented in Equation (8). In the initialization stage of the EKF, the a priori covariance matrix *P_k_* (−) is assumed to be a diagonal matrix that includes the variances of the state vector. The system noise covariance matrix *Q* is a diagonal matrix, which is furnished based on the noise covariance of each parameter in the system state.

(6) $$\delta x_{k}(-)=\phi_{k-1,k}\delta x_{k-1}(+)+G_{k-1}w$$

(7) $$\phi_{k-1,k}=F(t)\Delta t+I$$

(8) $$P_{k}(-)=\phi_{k,k-1}P_{k-1}(+)\phi_{k,k-1}^{T}+G_{k,k-1}Q_{k,k-1}G_{k,k-1}^{T}$$

where $\delta x_{k}\left(-\right)$ is the predicted state vector at epoch *k*, $\varphi_{k-1,k}$ is the state transition matrix from epoch *k* − 1 to *k*, $\delta x_{k-1}\left(+\right)$ is the estimated state vector to epoch *k* − 1, $G_{k-1}$ is the noise coupling matrix [22,23], w is the system noise with Q covariance matrix, *F*(*t*) is the system dynamic matrix, *Δt* is the sampling interval, *I* is the identity matrix, $P_{k-1}$ is the posteriori covariance matrix of the state vector at epoch *k* − 1, and $Q_{k,k-1}$ is the system noise covariance matrix. The error state of the system δx for the loosely coupled integration is represented by Equation (9).

(9) $$\delta x={\left[\begin{array}{ccccc} \delta r & \delta v & \delta \varepsilon & \delta b_{a} & \delta b_{g} \end{array}\right]}^{T}$$

where $\delta r={\left[\begin{array}{ccc} \delta \phi & \delta \lambda & \delta h \end{array}\right]}^{T}$ is the position error vector, $\delta v={\left[\begin{array}{ccc} \delta v_{e} & \delta v_{n} & \delta v_{u} \end{array}\right]}^{T}$ is the velocity error vector, $\delta \varepsilon ={\left[\begin{array}{ccc} \delta p & \delta r & \delta y \end{array}\right]}^{T}$ is the attitude angles’ error vector, $\delta b_{a}={\left[\begin{array}{ccc} \delta b_{ax} & \delta b_{ay} & \delta b_{az} \end{array}\right]}^{T}$ is the accelerometer bias error vector, and $\delta b_{g}={\left[\begin{array}{ccc} \delta b_{gx} & \delta b_{gy} & \delta b_{gz} \end{array}\right]}^{T}$ is the gyroscope bias error vector.

The system dynamic matrix is shown by Equation (13) [4]. It is worth noting that the accelerometer and gyroscope biases were modeled using a first-order Gauss–Markov (GM) process [9].

(10) $$F=\left[\begin{array}{ccccc} F_{rr} & F_{rv} & 0_{3\times 3} & 0_{3\times 3} & 0_{3\times 3}\\ F_{vr} & F_{vv} & F_{v\varepsilon } & R_{b}^{l} & 0_{3\times 3}\\ F_{er} & F_{\varepsilon v} & F_{ee} & 0_{3\times 3} & R_{b}^{l}\\ 0_{3\times 3} & 0_{3\times 3} & 0_{3\times 3} & F_{ba} & 0_{3\times 3}\\ 0_{3\times 3} & 0_{3\times 3} & 0_{3\times 3} & 0_{3\times 3} & F_{bg} \end{array}\right]$$

### 2.5. Measurement Model

The predictions of the EKF are modified during the update stage. The measurement model for the LC INS/LiDAR integration is presented by Equation (11).

(11) $$\delta Z_{k}=H_{k}\delta x_{k}+v_{k}$$

where $\delta Z_{k}$ is the measurement error vector, *H_k_* is the design matrix, $\delta x_{k}$ is the error state, and $v_{k}$ is the measurement noise. Since the EKF is operating on the error state of the system, the measurement update vector will be modeled as a measurement error vector as represented by Equation (12).

(12) $$\delta Z_{k}={\left[\begin{array}{cc} \delta r & \delta \varepsilon \end{array}\right]}^{T}={\left[\begin{array}{cccccc} \varphi_{imu-Li} & \lambda_{imu-Li} & h_{imu-Li} & p_{imu-Li} & r_{imu-Li} & y_{imu-Li} \end{array}\right]}^{T}$$

The design matrix *H* elements are depicted by Equation (13).

(13) $$H=\left[\begin{array}{ccccc} I_{3\times 3} & 0_{3\times 3} & 0_{3\times 3} & 0_{3\times 3} & 0_{3\times 3}\\ 0_{3\times 3} & 0_{3\times 3} & I_{3\times 3} & 0_{3\times 3} & 0_{3\times 3} \end{array}\right]$$

The covariance matrix of the measurements *R_k_* is assumed to be a diagonal matrix, including the variances of the position and attitude angles received from the LiDAR SLAM, which is depicted by Equation (14).

(14) $$R_{k}=diag\left\{\begin{array}{cccccc} \sigma_{\phi_{Li}}^{2} & \sigma_{\lambda_{Li}}^{2} & \sigma_{h_{Li}}^{2} & \sigma_{p_{Li}}^{2} & \sigma_{r_{Li}}^{2} & \sigma_{y_{Li}}^{2} \end{array}\right\}$$

where $\phi_{imu-Li}$, $\lambda_{imu-Li}$, $h_{imu-Li}$ are the measurement errors for the geodetic latitude, longitude and height; $p_{imu-Li}$, $r_{imu-Li}$, $y_{imu-Li}$ are the measurement errors for the pitch, roll and yaw angles; $\sigma_{\varphi_{Li}}^{2}$, $\sigma_{\lambda_{Li}}^{2}$, $\sigma_{h_{Li}}^{2}$ are the variances of the LiDAR geodetic latitude, longitude and height; and $\sigma_{p_{Li}}^{2}$, $\sigma_{r_{Li}}^{2}$, $\sigma_{y_{Li}}^{2}$ are the variances of the LiDAR geodetic pitch, roll and yaw angles. Subsequently, the Kalman gain *K_k_* and the posterior state covariance $P_{k}\left(+\right)$ are calculated using Equations (15) and (16).

(15) $$K_{k}=P_{k}(-)H_{k}^{T}{\left[H_{k}P_{k}(-)H_{k}^{T}+R_{k}\right]}^{-1}$$

(16) $$P_{k}(+)=(I-K_{k}H_{k})P_{k}(-){\left(I-K_{k}H_{k}\right)}^{T}+K_{k}R_{k}K_{k}^{T}$$

The innovation term $dx_{k}$ is calculated using Equation (17). However, since this study adopts a closed-loop error scheme, Equation (17) can be written in a more simplified format, as shown in Equation (18).

(17) $$dx_{k}=K_{k}\left[\delta z_{k}-H_{k}\delta x_{k}(-)\right]$$

(18) $$dx_{k}=K_{k}\delta z_{k}$$

Finally, the error state of the system can be updated using Equation (19), which can be further simplified using Equation (20).

(19) $$\delta x_{k}(+)=\delta x_{k}(-)+K_{k}\left[\delta z_{k}-H_{k}\delta x_{k}(-)\right]$$

(20) $$\delta x_{k}(+)=\delta x_{k}(+)+K_{k}\delta z_{k}$$

## 3. Data Source and Case Studies

The raw Karlsruhe Institute of Technology and Toyota Technological Institute (KITTI) dataset was considered in this study [24]. The KITTI dataset is an online dataset made available to all researchers worldwide. The KITTI data collection platform is a land vehicle equipped with several sensors, including an integrated GNSS/IMU unit (the OXTS RT3003 unit), a Velodyne HDL-64E mechanical LiDAR, a Sony greyscale stereo pair, and a Sony colored stereo pair. All the extrinsic and intrinsic calibration parameters of the system are estimated and provided with the dataset. The KITTI dataset can be divided into two main categories, namely the raw dataset and the odometry dataset. The raw dataset covers a wide range of driving environments in residential and highway areas, while the odometry dataset is obtained from the raw dataset for sequences of mostly residential areas [24]. Finally, the KITTI dataset provides the odometry benchmark [12] for researchers to showcase their work and the results of developing visual and LiDAR SLAM algorithms that satisfy certain conditions.

In this study, the raw KITTI data were adopted, from which three case studies were formulated to accommodate different driving environments and scenarios.

The first case study featured the use of residential raw KITTI datasets during a complete absence of GNSS signal along the whole trajectory of the vehicle. The residential datasets represent typical urban driving environments, where driving speed is relatively low to moderate, and the scene is feature-rich. Within the first case study, the datasets were broken down into three different scenarios: a sample relatively short dataset, a sample relatively long dataset, and the remaining residential KITTI datasets.

Similarly, the second case study followed the same structure as the first. However, it featured the KITTI road datasets, which represent highway driving environments. Furthermore, the scene in the highway environments included drastically fewer features than its urban counterpart, which was expected to adversely affect the performance of the LiDAR SLAM algorithm.

The third and final case study involved an attempt to improve the performance of the integrated INS/LiDAR SLAM navigation system in cases where the GNSS signal loss persists for prolonged periods of time, which leads to degradation in the performance of the integrated navigation system. Such improvement was studied by incorporating minimal assistance from the GNSS in the form of a single observation along the whole trajectory of the vehicle. The third case study was broken down into two different scenarios: a sample residential dataset and a sample highway dataset.

Descriptive information about the trajectories of the datasets used in all the previous three case studies and their corresponding scenarios are described in Table 1.

## 4. Analysis and Results

### 4.1. First Case Study—Residential Datasets (Complete GNSS Outage)

The first case study mimicked a full GNSS signal outage along the whole trajectory of the vehicle while driving in urban environments. In this case study, the raw KITTI residential datasets were adopted. Furthermore, the datasets were further categorized into three categories: a short sample dataset, a long sample dataset, and other residential datasets.

For all the case studies, the ground truth was the integrated solution of the OXTS GNSS/INS system used in the KITTI data collection platform, whose measurements were provided in the raw KITTI dataset [24]. The position and attitude solutions of the integrated INS/LiDAR SLAM navigation system were compared to the ground truth for all cases.

#### 4.1.1. Sample Relatively Short Residential Dataset

**Figure 3:**
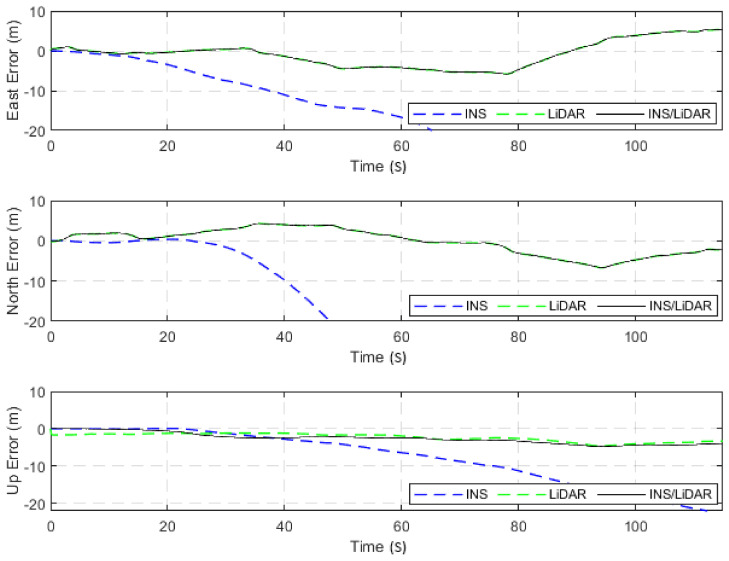
D-27—complete GNSS signal outage: position errors (ENU).

**Figure 4:**
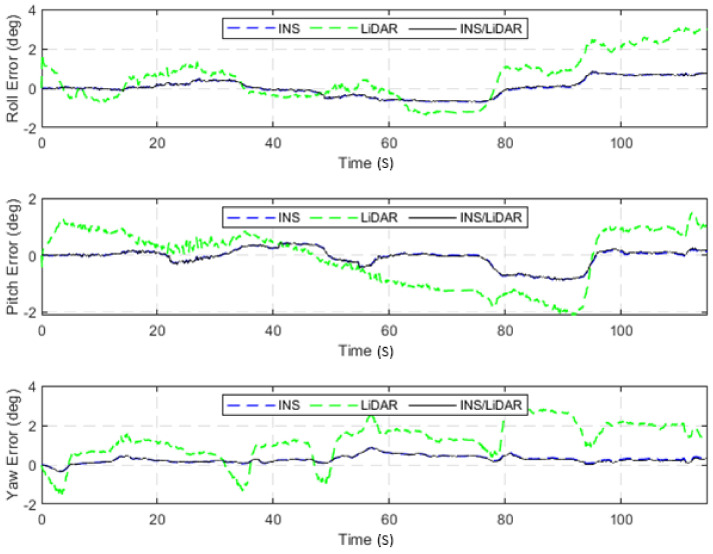
D-27—complete GNNS signal outage: errors of attitude angles (roll, pitch, and yaw).

The dataset used in this scenario was 2011_09_30_drive_0027_sync, labelled as D-27. This was a 692.47-m long drive, lasting 114.85 s at an average speed of 21.71 km/h. Figure 3 illustrates the position errors in the ENU local frame, while Figure 4 presents the errors of the attitude angles (roll, pitch, and yaw) for three navigation solutions. The first used the full INS-only solution without any update from the LiDAR SLAM. The second navigation solution was obtained through the LIDAR SLAM only. Finally, the third navigation solution was the integrated INS/LiDAR SLAM system. These figures are quantified in Table 2, where the position and attitude error statistics are shown for the three navigation solutions.

It is noticeable from Figure 3 that there was a large drift of the INS solution over time, which indicates that it cannot be used as the sole system for precise navigation. However, the integrated INS/LiDAR navigation solution exhibited significantly less error in comparison with the INS solution. Therefore, the integrated INS/LiDAR solution was tuned such that it emphasized the weights of position updates from the LiDAR SLAM, while de-emphasizing that of the INS solution. As a result, the integrated INS/LiDAR position solution was closer to the LiDAR SLAM solution.

**Figure 5:**
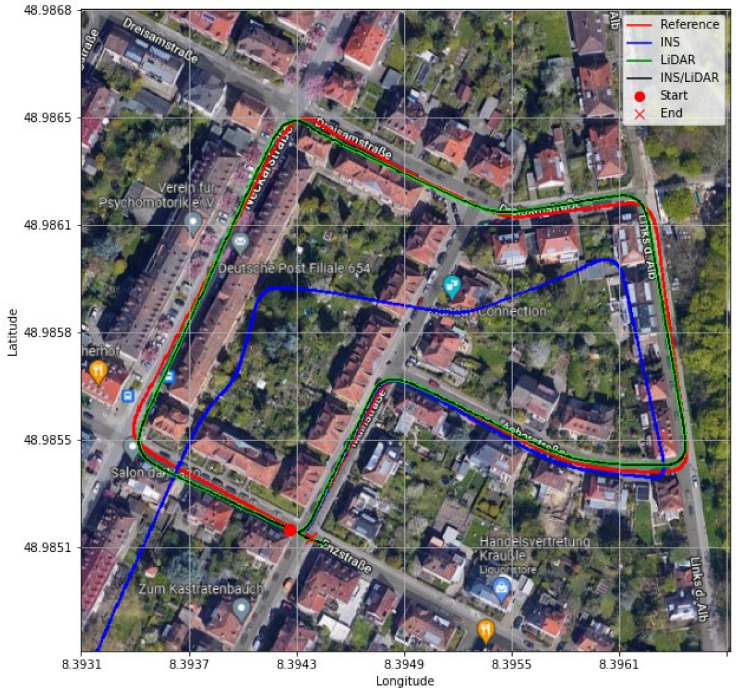
D-27—complete GNNS signal outage: comparison of trajectories.

In contrast, in Figure 4, the integrated INS/LiDAR solution was tuned to follow the INS solution for the attitude angles, which outperformed that of the LiDAR SLAM. The reason for this was that the IMU directly measured the vehicle’s accelerations and angular rotations, after which the attitude was estimated. By contrast, the LiDAR estimates of the attitude angles were obtained through the SLAM solution (point cloud registration), which led to a significant amount of error accumulation. These trends are numerically noticeable in Table 2, where the RMSE of the integrated INS/LiDAR position errors converged towards that of the LiDAR SLAM. By contrast, the RMSE of the attitude errors was closer to the mechanization errors. All trajectories were compared to the ground truth, which was the integrated solution of the OXTS unit provided in the raw KITTI dataset. The trajectories for the complete outage of the residential dataset are presented in Figure 5, where the mechanization, LiDAR SLAM, and INS/LiDAR trajectories are compared to the ground truth.

#### 4.1.2. Sample Relatively Long Residential Dataset

**Figure 6:**
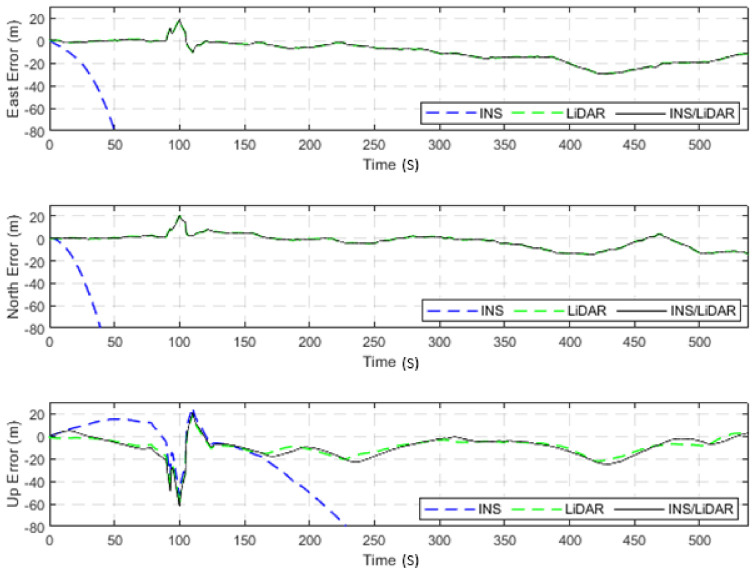
D-28—complete GNSS signal outage: position errors (ENU).

**Figure 7:**
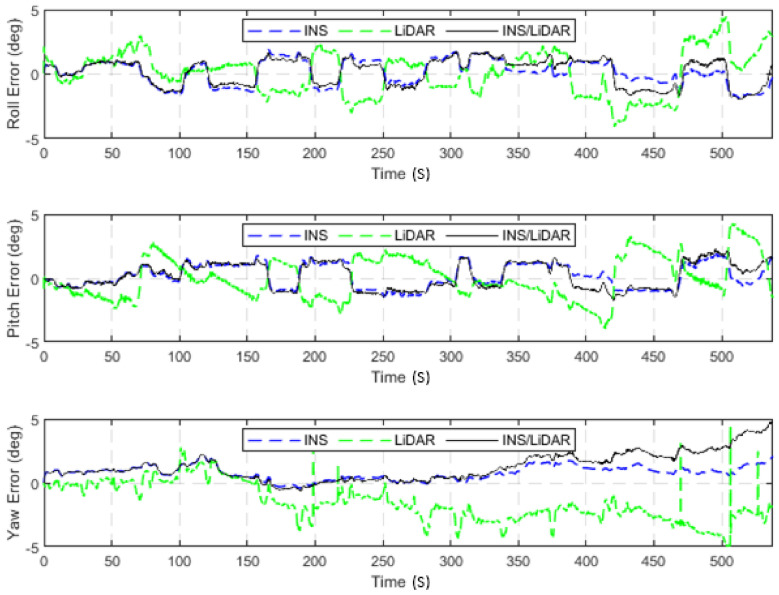
D-28—complete GNNS signal outage: errors of attitude angles (roll, pitch, and yaw).

In this scenario, a significantly longer dataset, 2011_09_30_drive_0028_sync, labelled as D-28, was considered. This is an approximately 9-min drive with an average speed of 28.17 km/h. Similar to the previous scenario, the same analysis was performed for this drive. Figure 6 and Figure 7 present the position and attitude errors, respectively. Table 3 shows the statistical characteristics of the position and attitude errors of D-28.

**Figure 8:**
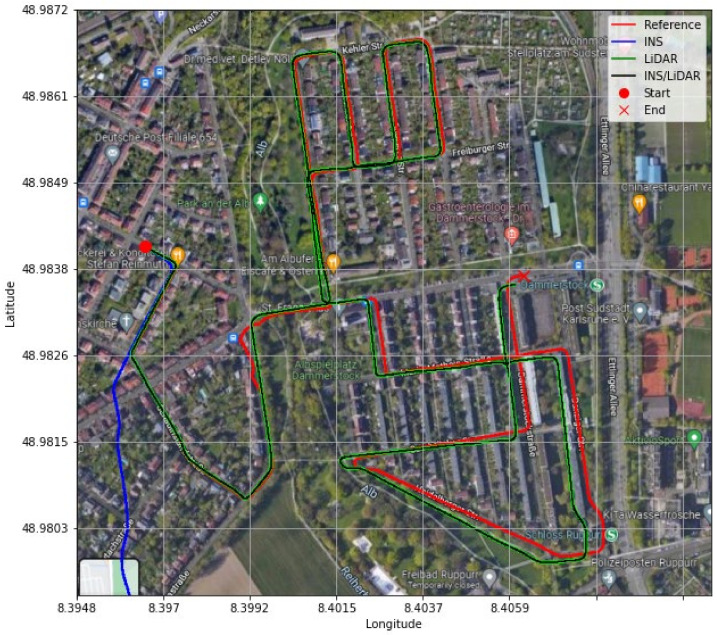
D-28—complete GNNS signal outage: comparison of trajectories.

It is noticeable from Figure 6 that the INS position solution deteriorated significantly in all directions. The drift rate was much higher than the previous scenario in D-27 because D-28 was significantly longer. This is reflected numerically in Table 3 for the INS navigation solution statistics. However, the INS still succeeded in yielding a more stable solution for the attitude angles, even for the longer dataset. While this may seem counter-intuitive, because the INS solution was prone to drift for both position and attitude estimates, the reason for the better performance was the quality of the IMU used in the KITTI data collection platform (OXTS unit). Furthermore, as mentioned in the discussion of the previous scenario, the IMU measures the attitude angles in a more direct way than the LiDAR SLAM. Through a careful examination of the IMU mechanization equations [4], the IMU measures the attitude angles in relation to the inertial system, from which the attitude angles can be estimated in the local-level reference frame. The attitude angles evolve from one epoch to the subsequent one using the updated quaternion. This led to less accumulated error over time, given that the used IMU was high-grade rather than of low-cost. Conversely, the attitude angles resulting from the LiDAR SLAM algorithms depended on the quality of the point-cloud-matching in a pair-wise fashion, which led to significant error accumulation while processing a stream of point clouds. Figure 8 presents a comparison between the trajectories of the INS, LiDAR, and INS/LiDAR algorithms versus the ground truth for D-28.

#### 4.1.3. KITTI Residential Datasets

**Figure 9:**
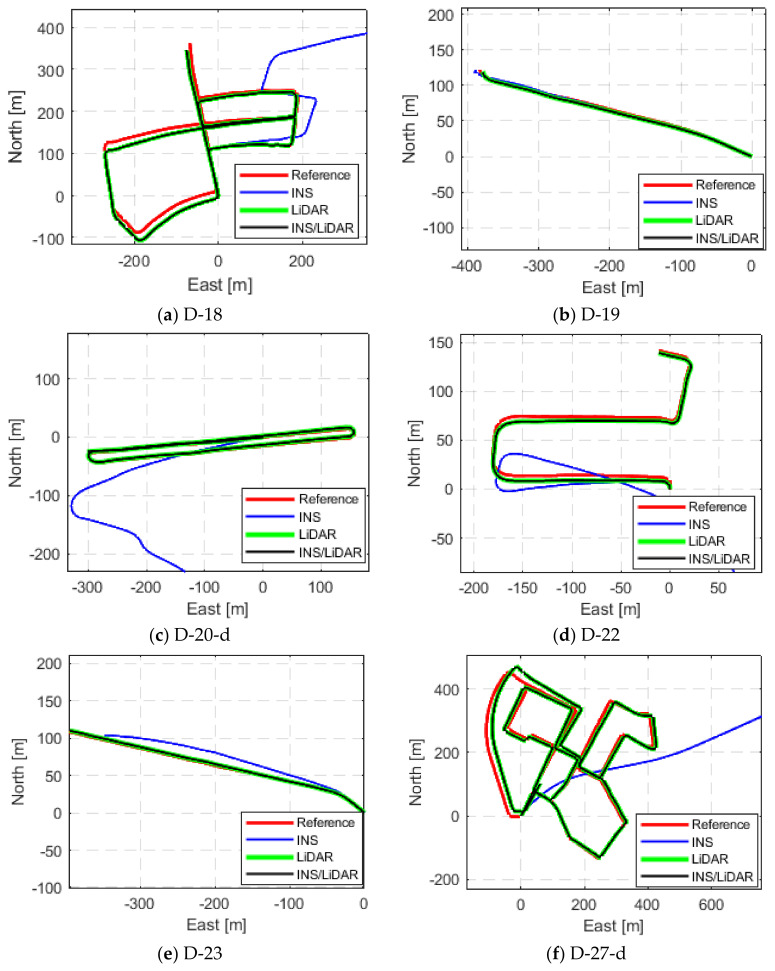
Complete GNNS signal outage: comparison of trajectories (D-18 to D-27_d).

**Figure 10:**
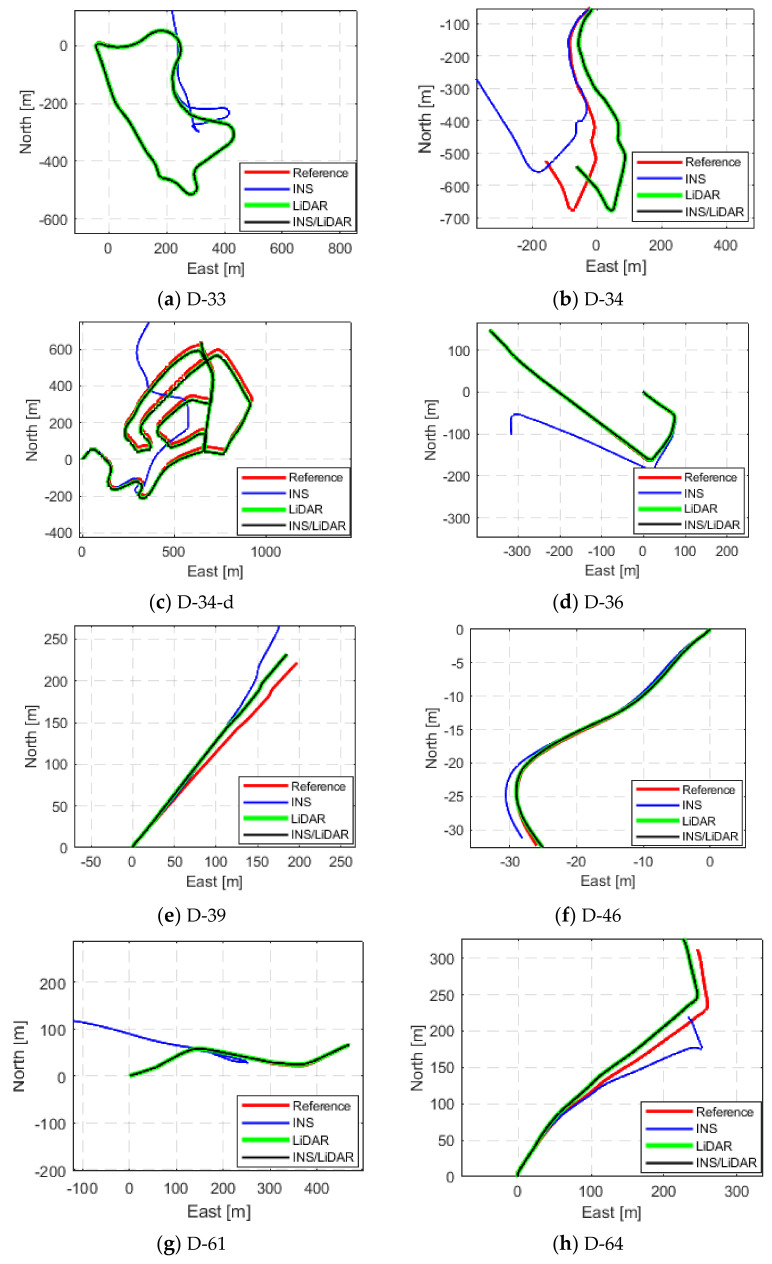
Complete GNNS signal outage: comparison of trajectories (D-33 to D-64).

**Figure 11:**
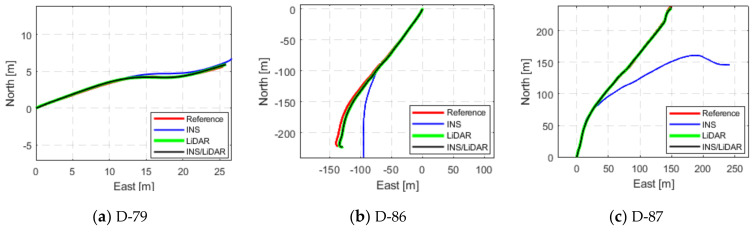
Complete GNNS signal outage: comparison of trajectories (D-79 to D-87).

The third and final scenario in this first case study involved showcasing the results of the proposed integrated navigation system for several of the raw KITTI datasets. This is depicted in Figure 9, Figure 10 and Figure 11, where the navigation solutions of the INS, LiDAR SLAM, and INS/LiDAR are compared to the ground truth for every dataset.

**Figure 12:**
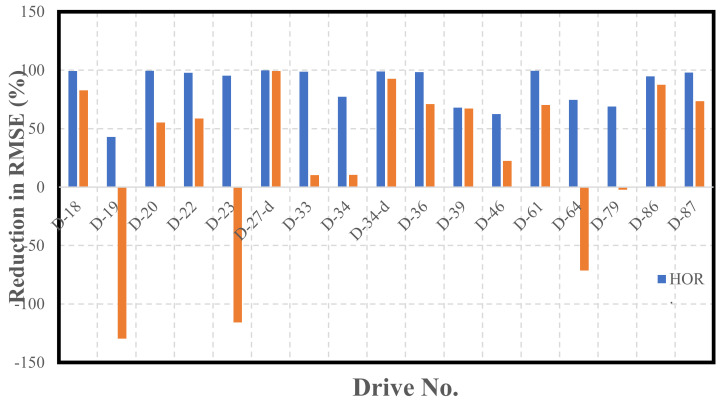
Reductions in RMSE of KITTI residential datasets.

Figure 12 presents a thorough evaluation of the developed integrated INS/LiDAR navigation performance using the datasets in this case study. The figure graphically depicts the reductions in the RMSE as a result of using the proposed LiDAR/INS integrated navigation system as opposed to its INS counterpart. Additionally, Table A1, Table A2, Table A3, Table A4, Table A5, Table A6, Table A7, Table A8, Table A9, Table A10, Table A11, Table A12, Table A13, Table A14, Table A15, Table A16 and Table A17 presented in Appendix A show the detailed results of the position and attitude error statistics.

Figure 9, Figure 10 and Figure 11 reinforce the conclusion that the integrated INS/LiDAR SLAM navigation system performed significantly better than the INS for position estimation. However, there were some cases where it was observable that the INS performance was almost as accurate as the integrated navigation system, which was exemplified by drives D-19 and D-79. Additionally, it is observable from Figure 12 that, for most drives, the proposed integrated navigation system outperformed the INS position estimation. However, for drives D-19, D-23, and D-64, the INS outperformed the integrated system for the up-direction estimates. The reason for this was that the datasets were shorter in length and time with minimal turns in the E-N plane (almost straight lines). Therefore, for these reasons and because of the relatively high grade of the OXTS IMU, the total drift was low.

### 4.2. Second Case Study—Highway Datasets (Complete GNSS Outage)

The second case study featured driving in highway environments. When using the LIDAR SLAM algorithms, one of the main challenges is feature extraction and feature matching among consecutive point cloud frames. Therefore, highway driving is challenging.

The proposed integrated navigation system was evaluated in such a challenging environment using different scenarios by testing several datasets of different lengths from the raw KITTI highway dataset.

#### 4.2.1. Sample Relatively Short Highway Dataset

**Figure 13:**
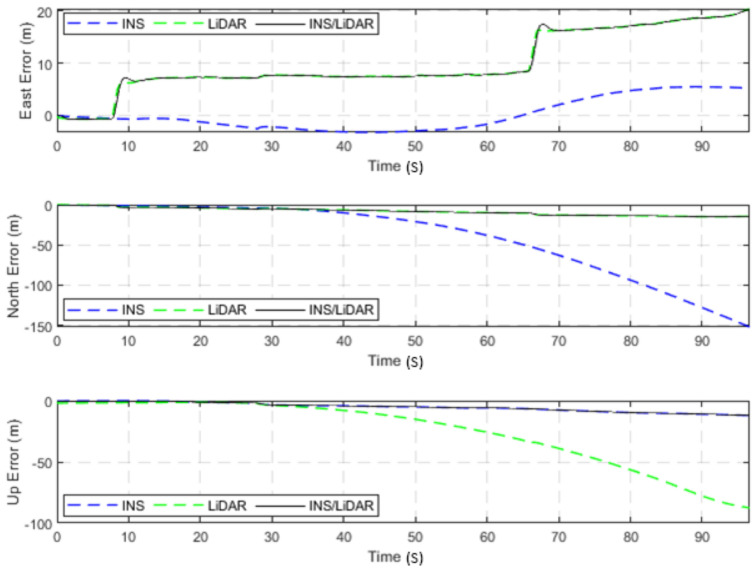
D-101—complete GNSS signal outage: position errors (ENU).

**Figure 14:**
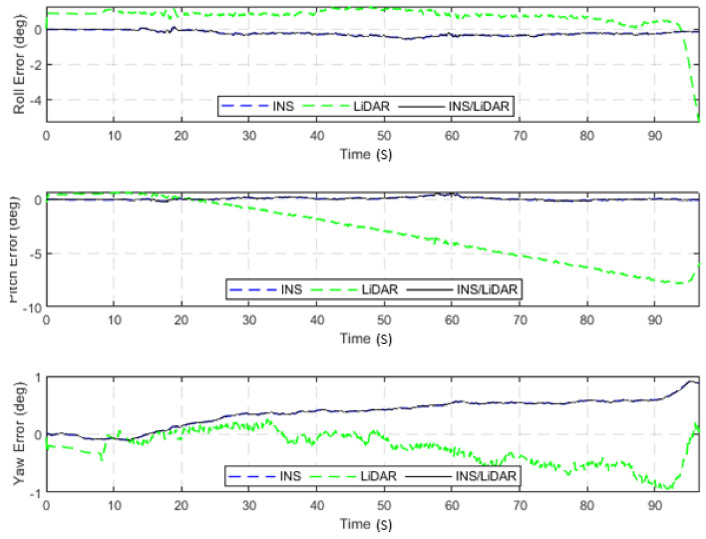
D-101—complete GNNS signal outage: errors of attitude angles (roll, pitch, and yaw).

In the first scenario, D-101 was considered, which represents a relatively short dataset. This dataset involved a length of 1299.13-m and a 96.62-s driving time with an average speed of 48.40 km/h. Figure 13 and Figure 14 show the position and attitude errors, respectively, while Table 4 quantifies these errors statistically.

As shown in Figure 13, overall, the integrated INS/LiDAR outperformed the INS solution, even though the INS yielded better position estimates in the east direction. This was because the INS drifted significantly in the north direction. As a result, the net horizontal position was in favor of the integrated navigation system. This is noticeable numerically from the values presented in Table 4. In Figure 14, similar to the residential datasets in the first case study, the results show that the INS attitude estimations outperformed their LiDAR counterpart.

**Figure 15:**
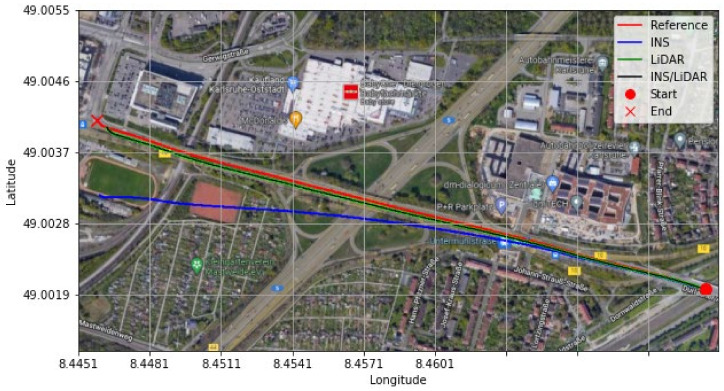
D-101—complete GNNS signal outage: comparison of trajectories.

Figure 15 illustrates the resultant trajectories of the three navigation solutions versus the ground truth trajectory for D-101.

#### 4.2.2. Sample Relatively Long Highway Dataset

**Figure 16:**
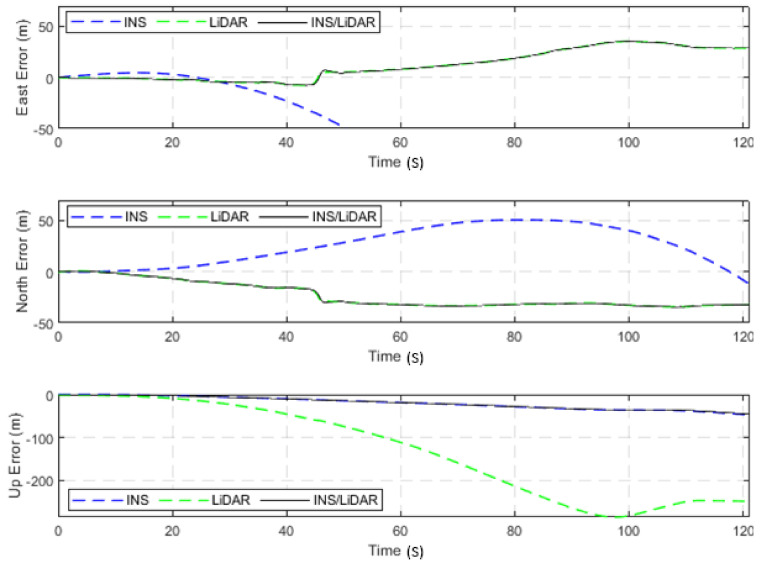
D-42—complete GNSS signal outage: position errors (ENU).

**Figure 17:**
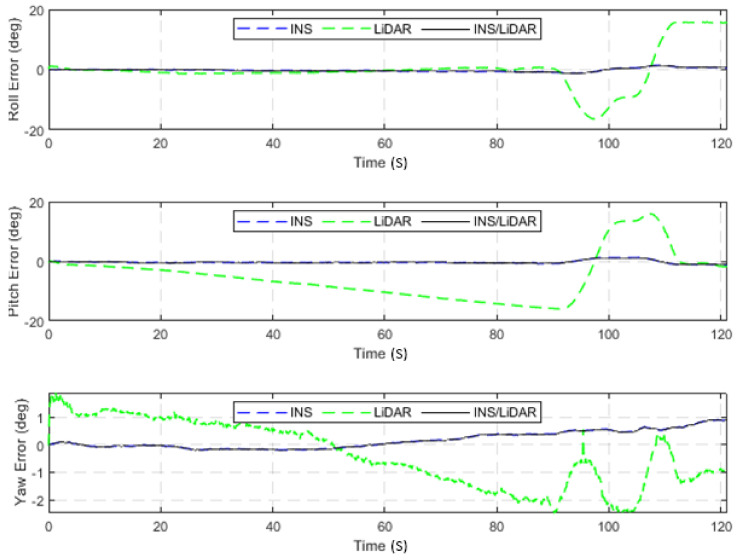
D-42—complete GNNS signal outage: errors of attitude angles (roll, pitch, and yaw).

In the second scenario of this case study, the integrated INS/LiDAR SLAM navigation system was tested using a relatively long highway drive of the raw KITTI dataset, 2011_10_03_drive_0042_sync, labelled as D-42. The drive involves travel along an approximately 2.6-km highway segment in 121.19 s at an average speed of approximately 77 km/h, which is the longest highway dataset in the raw KITTI datasets. The position and attitude errors of D-42 are presented in Figure 16 and Figure 17, respectively, while Table 5 quantifies these errors numerically for all resultant navigation solutions.

Similar to the previous scenario in the second case study, from Figure 16 and Figure 17, the same conclusions are reinforced, that the LiDAR SLAM outperformed the INS for position estimations, albeit not for the attitude estimation.

**Figure 18:**
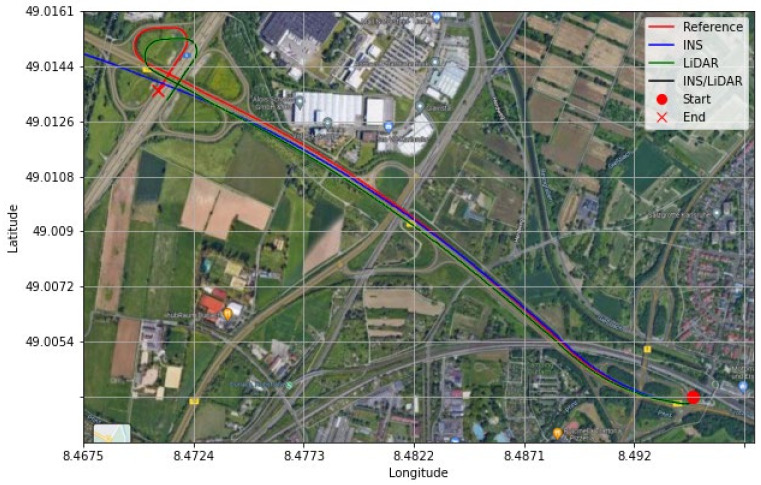
D-42—complete GNNS signal outage: comparison of trajectories.

The INS, LiDAR SLAM, and INS/LiDAR trajectories of D-42 are compared against the reference trajectory in Figure 18.

#### 4.2.3. KITTI Highway Datasets

**Figure 19:**
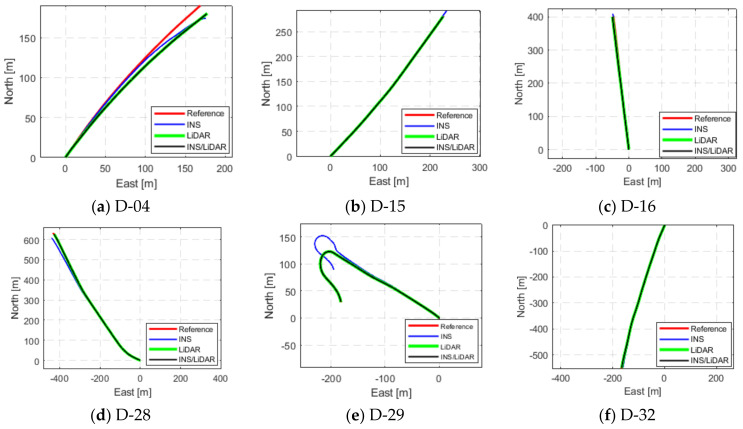
Complete GNNS signal outage: comparison of trajectories.

In the third and final scenario of the second case study, a combination of different highway raw KITTI datasets was used to test the proposed integrated navigation system. Figure 19 presents the results of comparing the navigation solution trajectories to the ground truth trajectory for all the datasets considered.

**Figure 20:**
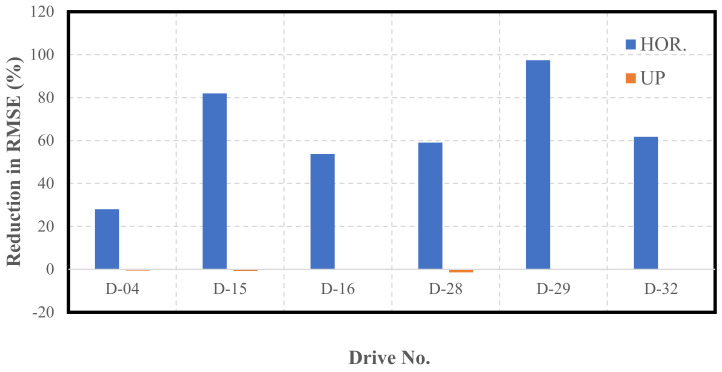
Reductions in RMSE of KITTI highway datasets.

Figure 20 illustrates a graphical summary of the reduction in the RMSE values as a result of using the proposed integrated navigation system as opposed to sole use of the INS. The results are presented in detail in Table A18, Table A19, Table A20, Table A21, Table A22 and Table A23, as attached in Appendix A.

It is noticeable from Figure 20 that the overall performance of the LiDAR SLAM, and, thereby, the INS/LiDAR SLAM integrated system was significantly better than the INS, especially for the net horizontal positioning estimation. However, there was only a slight improvement over the averages in the up direction. In contrast, the improvements in the up-direction for the residential datasets were significantly greater, as shown in Figure 12. This was potentially due to the way the up direction was estimated in comparison with the estimations for the east and north directions. That is, by nature, the collected point clouds included points that were very well distributed around the LiDAR scanner from all directions, such that the LiDAR was centered among the points. However, the vast majority of the collected points were above the LiDAR sensor, which meant that very few points, to almost none, were below the level of the LiDAR sensors. As a result, the points were poorly distributed along the vertical direction, which led to poor geometry. This is analogous to GNSS positioning, where the horizontal dilution of precision (HDOP) is always lower than the vertical dilution of precision (VDOP), due to the poor geometry of the satellites around the GNSS receiver in the vertical direction [25].

Based on the above analysis, the LiDAR SLAM appears to be highly sensitive in the up-direction, such that many features are needed to obtain a good estimation of the up-direction. This is why it is evident that residential, feature-rich environments (as shown in Figure 12) provide better estimations of the up-direction as opposed to highway, feature-poor environments (as shown in Figure 20).

Additionally, comparing the results of the up component considering Figure 12 (residential datasets) and Figure 20 (highway datasets), it can be concluded that the performance of the LiDAR SLAM algorithm in the up direction was significantly better for feature-rich residential areas.

It is essential to note that the improvements presented in Figure 20 are in the form of percentages. This does not necessarily reflect the visual comparison of the trajectories provided in Figure 19. For example, D-15 showed an improvement in the horizontal positioning direction of just over 80%; however, this is not visually present in Figure 19b. This is because the drive length was too short; therefore, the numerical quantification is of the utmost importance to avoid the drawing of any misleading conclusions.

It is important to note that the performance of the LiDAR SLAM in the case of the highway dataset was generally poorer when compared to its performance in the first case study. This was essentially due to the different nature of the driving environment and the sparseness of the highway scene in terms of the availability of features in comparison with the urban scenes.

### 4.3. Third Case Study—GNSS Assistance

The third case study considered improvement in the performance of the proposed integrated navigation system in cases where the GNSS signal outages persist for prolonged periods of time. This was accomplished by assuming minimal assistance from the GNSS, mimicking the intermittent receipt and outage of GNSS signals. An analogy to this scenario is driving into an urban canyon with high-rise buildings and skyscrapers which almost completely block the GNSS signal.

Two scenarios were considered in this case study. The first involved the use of a long, residential dataset (D-28), which was the same as the one used in the first case study (second scenario). Similarly, the second scenario considered the effect of GNSS assistance using one of the highway datasets of the KITTI datasets (D-42), which was previously considered in the second case study (second scenario).

#### 4.3.1. Sample Residential Dataset (GNSS-Assisted)

**Figure 21:**
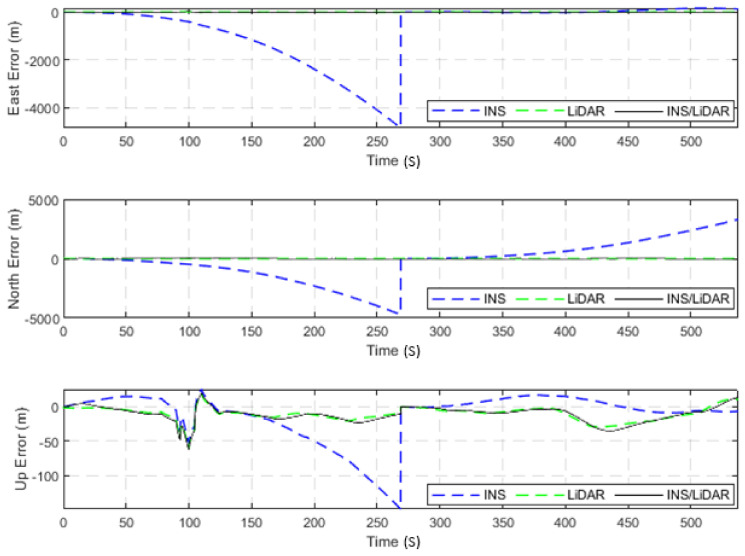
D-28—GNSS-assisted: position errors (ENU).

**Figure 22:**
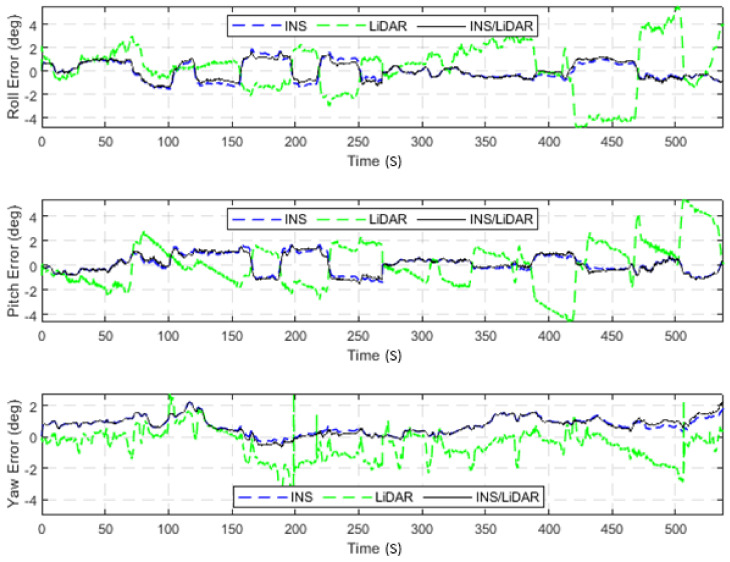
D-28—GNSS-assisted: errors of attitude angles (roll, pitch, and yaw).

The first scenario involved the nearly 9-min drive (D-28) of the residential raw KITTI drives. A complete GNSS signal outage was assumed along the whole trajectory of the vehicle. However, a simulated single update from the GNSS was received after half the time of the drive had elapsed—at approximately the 4.5-min mark. Figure 21 and Figure 22 show the position and attitude errors, respectively, for D-28 with assistance from GNSS. The errors are presented numerically and statistically in Table 6.

**Figure 23:**
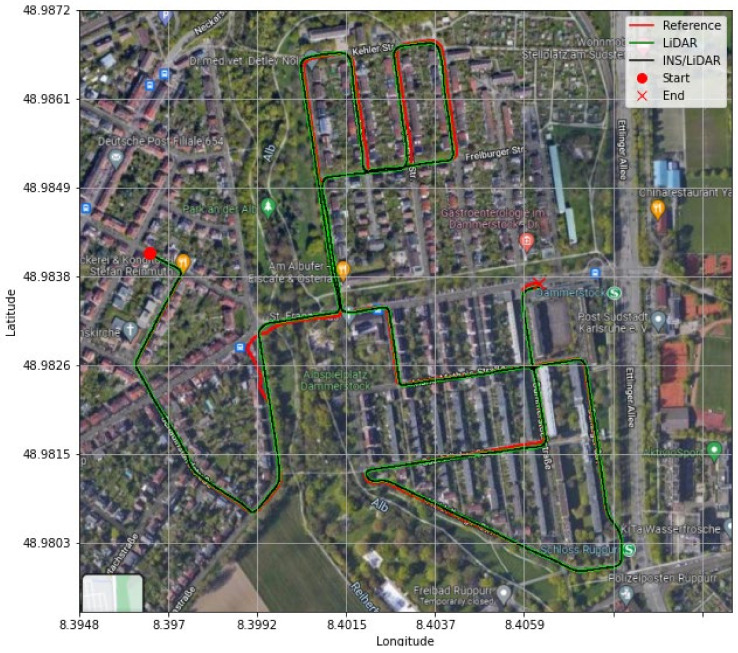
D-28—GNNS assisted: comparison of trajectories.

It is evident from Figure 21 and Figure 22 that a significant improvement in both position and attitude estimations was accomplished by a single GNSS update at the middle point of the trajectory. This is further illustrated in Table 6, where the RMSE for the whole trajectory was reduced to 4.96 m for the horizontal direction. To emphasize the significance of such an improvement, for the same drive D-28 presented in the first case study, the RMSE value for the horizontal direction was 15.06 m. The results are represented visually on the base map shown in Figure 23, where the three navigation solutions of D-28 (GNSS-assisted) are shown versus the ground truth trajectory.

#### 4.3.2. Sample Highway Dataset (GNSS-Assisted)

**Figure 24:**
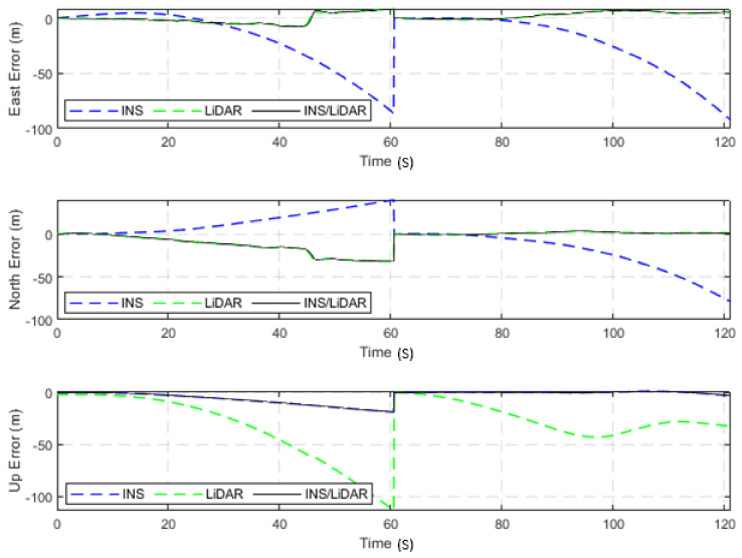
D-42—GNSS-assisted: position errors (ENU).

**Figure 25:**
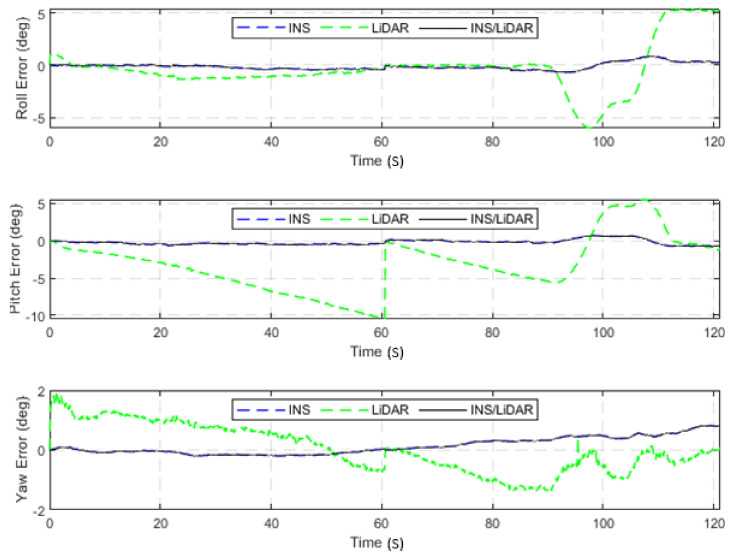
D-42—GNSS-assisted: errors of attitude angles (roll, pitch, and yaw).

The second and final scenario in this case study involved the use of D-42, which represents the use of a relatively long highway drive from the raw KITTI dataset. Similar to the previous scenario, only one GNSS update was received at the middle point of the drive—approximately the 39-s mark. The position and attitude errors of D-42 are presented in Figure 24 and Figure 25, respectively, while Table 7 shows the statistical characteristics of these errors.

**Figure 26:**
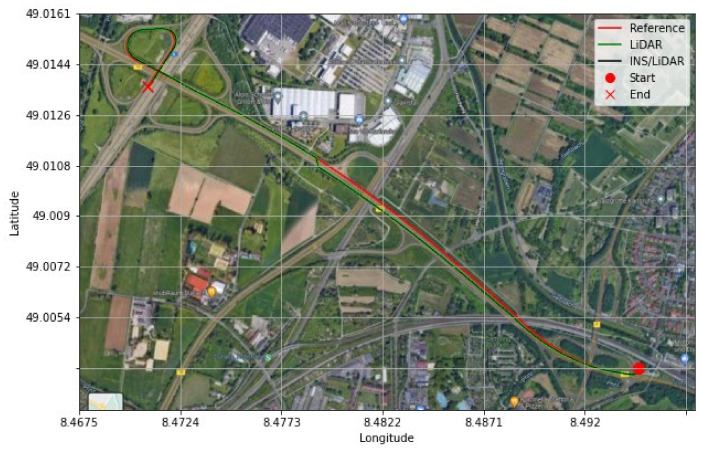
D-42—GNNS-assisted: comparison of trajectories.

Figure 24 and Figure 25 illustrate the improvements in the position and attitude of D-42 in comparison with Figure 16 and Figure 17 for the same dataset in the case of an artificial complete GNSS signal outage. Similarly, the positioning results show significant improvements, as shown in Table 7, when compared to the results shown in Table 5, where the horizontal position RMSE was reduced from 32.15 m to 13.19 m. Figure 26 shows the resulting three navigation solutions of D-42 versus the ground truth trajectory.

## 5. Comparison to State-Of-The-Art SLAM Algorithms

The proposed INS/LiDAR integrated navigation system was compared to three other state-of-the-art LiDAR SLAM algorithms, namely A-LOAM [13,26], LeGO-LOAM [14,27], and F-LOAM [17,28]. These SLAM algorithms were used to process three datasets, namely D-28 (residential), D-42 (highway), and D-101 (highway). Since the performance of SLAM algorithms can be sensitive to the machine performance, it is important to note that these algorithms were installed and tested on a machine with the following specifications and environment configurations: ROS Melodic—Ubuntu 18.05 (Intel^®^ core™ i7-8550U CPU @ 1.80 GHZ and 16 GB RAM).

**Figure 27:**
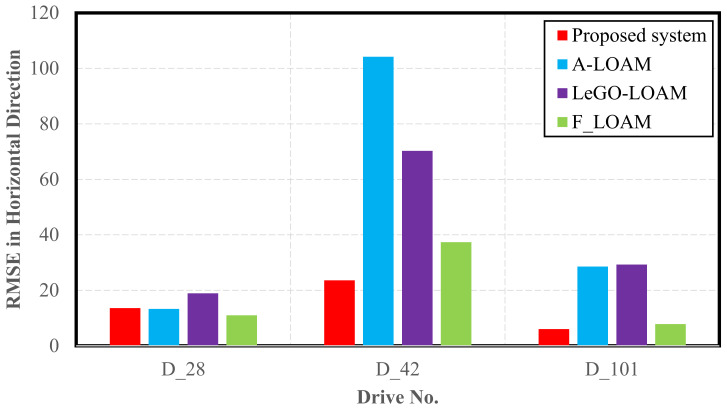
Performance comparison between the proposed integrated system and state-of-the-art SLAM algorithms (RMSE in horizontal direction).

As shown in Table 8 and Figure 27, the proposed integrated INS/LiDAR system slightly outperformed the A-LOAM and Le-GO LOAM algorithms for the residential drive D-28. In addition, the system produced almost equal results for the RMSE compared to the F-LOAM. This was expected, as residential areas are characterized by being feature-rich, which simplifies and enhances the accuracy of SLAM algorithms. However, for the highway datasets (D-42 and D-101), the system significantly outperformed all three algorithms.

## 6. Conclusions

In this paper, an LC integrated navigation system was proposed to fuse the INS and the LiDAR SLAM (Kitware) algorithms using an EKF. The proposed navigation system was tested for various driving environments and scenarios using the raw residential and highway KITTI datasets. Three case studies were presented. One featured the use of residential datasets of the raw KITTI datasets, which involved a total driving time of 48 min for all the considered datasets during a complete artificial outage of the GNSS signal. For all the scenarios, the LiDAR SLAM system outperformed the INS for position estimation in all directions. However, for short datasets (below 1 min), the INS performed as well as the LiDAR SLAM system due to the high quality of the IMU (OXTS) of the KITTI data collection platform, which made it less prone to drift for short periods of time. In contrast, the INS yielded better attitude estimations than the LiDAR SLAM system.

The second case study examined the use of highway datasets for a total time of 7 min, which comprised all the available highway datasets in the raw KITTI data. Similar to the first case study, the LiDAR SLAM system yielded better positioning results for the longer datasets. However, the INS yielded better results for the up direction, unlike for the first case study. This was because the LiDAR SLAM is very sensitive when estimating the up-direction in comparison to the north and east directions due to the poor geometry of the point cloud distribution in the vertical direction. Therefore, the more the extracted features, the better the up-direction estimation.

The INS also outperformed the LiDAR SLAM for attitude estimation. Although the LiDAR SLAM system produced satisfactory results for the highway datasets, its performance was better in the case of driving in residential areas. This was because residential environments are feature-rich.

The third and final case study involved driving within residential and highway environments where intermittent GNSS signal outages occurred, mimicking driving through urban canyons and highways with tunnels. The results for both the residential and highway datasets showed significant improvement in comparison with the same datasets in the first case study, where a complete artificial outage of the GNSS signal was involved.

For all the driving scenarios, the integrated INS/LiDAR SLAM navigation system yielded positioning results for the residential datasets that outperformed the INS by an average RMSE reduction of 88% and 32% in the horizontal and up components, respectively. For the highway datasets, the improvements were of the order of 70% and 0.2% for the horizontal and up components, respectively.

The proposed system was compared to three state-of-the-art LiDAR SLAM algorithms. The system yielded slightly better performance than its counterparts for residential datasets, but significantly outperformed other SLAM algorithms for highway environments.

## Figures and Tables

**Table 1:** Trajectory information for the KITTI datasets considered [24].

Drive Label	Drive Number	Length (m)	Time (s)	Average Speed (km/h)	No. of Frames
	Residential datasets				
D-18	2011_09_30_drive_0018_sync	2206.47	287.53	27.63	2761
D-19	2011_09_26_drive_0019_sync	406.48	49.61	29.50	480
D-20	2011_09_30_drive_0020_sync	1233.74	114.49	38.79	1104
D-22	2011_09_26_drive_0022_sync	515.17	82.67	22.43	799
D-23	2011_09_26_drive_0023_sync	413.99	48.91	30.47	473
D-27	2011_09_30_drive_0027_sync	692.47	114.85	21.71	1106
D-27-d	2011_10_03_drive_0027_sync	3669.18	465.97	28.35	4497
D-28	2011_09_30_drive_0028_sync	4208.65	537.78	28.17	5177
D-33	2011_09_30_drive_0033_sync	1709.57	165.31	37.23	1594
D-34	2011_09_30_drive_0034_sync	920.52	126.88	26.12	1224
D-34-d	2011_10_03_drive_0034_sync	5043.67	481.31	37.72	4642
D-36	2011_09_26_drive_0036_sync	715.57	82.68	31.16	802
D-39	2011_09_26_drive_0039_sync	297.78	40.57	26.42	394
D-46	2011_09_26_drive_0046_sync	47.56	12.81	13.37	125
D-61	2011_09_26_drive_0061_sync	494.02	72.61	24.49	703
D-64	2011_09_26_drive_0064_sync	437.89	58.74	26.84	569
D-79	2011_09_26_drive_0079_sync	26.27	10.24	9.23	100
D-86	2011_09_26_drive_0086_sync	268.72	72.84	13.28	705
D-87	2011_09_26_drive_0087_sync	286.79	75.27	13.72	728
	Highway datasets				
D-04	2011_09_29_drive_0004_sync	255.05	35.15	26.13	339
D-15	2011_09_26_drive_0015_sync	362.81	30.65	42.61	297
D-16	2011_09_30_drive_0016_sync	404.71	28.84	50.52	278
D-28	2011_09_26_drive_0028_sync	779.50	44.43	63.16	430
D-29	2011_09_26_drive_0029_sync	351.24	44.46	28.44	430
D-32	2011_09_26_drive_0032_sync	578.30	40.31	51.64	390
D-42	2011_10_03_drive_0042_sync	2591.80	121.19	76.99	1170
D-101	2011_09_26_drive_0101_sync	1299.13	96.62	48.40	936

**Table 2:** D-27—position (m) and attitude (deg) error statistics.

	INS			LiDAR			INS/LiDAR		
	Mean	RMSE	Max	Mean	RMSE	Max	Mean	RMSE	Max
East	−30.38	44.27	122.57	−0.88	3.34	5.84	−0.88	3.34	5.84
North	−88.24	136.01	353.70	−0.22	3.12	6.71	−0.22	3.12	6.71
Horizontal	94.16	143.03	374.34	4.22	4.57	7.19	4.22	4.57	7.19
Up	7.80	10.62	22.85	2.31	2.55	4.57	2.57	2.95	4.78
Roll	0.012	0.444	0.838	0.504	1.276	3.084	0.022	0.441	0.852
Pitch	−0.076	0.332	0.885	−0.098	0.989	2.123	−0.073	0.330	0.881
Yaw	0.280	0.337	0.863	1.233	1.555	3.027	0.262	0.322	0.868

**Table 3:** D-28—position (m) and attitude (deg) error statistics.

	INS			LiDAR			INS/LiDAR		
	Mean	RMSE	Max	Mean	RMSE	Max	Mean	RMSE	Max
East	−7196.25	10,042.85	23,518.43	−9.94	13.57	29.68	−9.94	13.57	29.68
North	−6188.34	8316.52	17,618.60	−2.29	6.53	20.69	−2.29	6.53	20.70
Horizontal	9500.92	13,039.30	29,385.90	12.09	15.06	31.86	12.09	15.06	31.86
Up	252.12	367.13	893.97	9.99	12.46	60.66	10.61	13.57	62.23
Roll	0.111	0.956	1.926	0.085	1.675	4.495	0.167	1.036	1.968
Pitch	0.171	0.937	2.032	0.020	1.647	4.326	0.157	1.025	2.304
Yaw	0.802	0.963	2.213	−1.556	2.146	5.159	1.247	1.670	4.797

**Table 4:** D-101—position (m) and attitude (deg) error statistics.

	INS			LiDAR			INS/LiDAR		
	Mean	RMSE	Max	Mean	RMSE	Max	Mean	RMSE	Max
East	0.07	3.06	5.48	9.93	11.43	20.46	9.93	11.44	20.42
North	−40.20	60.39	152.14	−8.23	9.40	14.95	−8.24	9.42	14.95
Horizontal	40.42	60.46	152.22	13.07	14.80	25.10	13.08	14.82	25.03
Up	4.89	6.06	11.72	25.00	36.65	87.50	4.86	6.03	11.66
Roll	−0.234	0.273	0.567	0.733	0.998	5.318	−0.234	0.273	0.568
Pitch	0.057	0.145	0.558	−2.995	4.092	7.882	0.057	0.145	0.558
Yaw	0.375	0.444	0.911	−0.238	0.368	0.965	0.375	0.444	0.911

**Table 5:** D-42—position (m) and attitude (deg) error statistics.

	INS			LiDAR			INS/LiDAR		
	Mean	RMSE	Max	Mean	RMSE	Max	Mean	RMSE	Max
East	−155.14	232.32	600.40	11.88	18.67	35.49	11.85	18.65	35.49
North	25.46	31.75	50.88	−23.10	26.17	34.33	−23.11	26.19	34.36
Horizontal	160.79	234.48	600.52	27.86	32.15	48.39	27.86	32.15	48.40
Up	19.48	24.09	47.33	130.98	167.81	285.49	19.15	23.58	45.66
Roll	−0.167	0.569	1.421	−0.045	6.294	16.392	−0.166	0.569	1.420
Pitch	−0.314	0.613	1.210	−5.189	9.368	16.017	−0.315	0.613	1.209
Yaw	0.172	0.355	0.903	−0.345	1.221	2.483	0.170	0.352	0.897

**Table 6:** D-28—GNSS-assisted—position (m) and attitude (deg) error statistics.

	INS			LiDAR			INS/LiDAR		
	Mean	RMSE	Max	Mean	RMSE	Max	Mean	RMSE	Max
East	−687.00	1423.76	4839.25	−0.25	3.64	18.39	−0.25	3.64	18.39
North	−194.20	1710.08	4720.22	0.23	3.36	20.69	0.23	3.36	20.70
Horizontal	1504.87	2225.19	6760.09	3.83	4.96	27.69	3.83	4.96	27.69
Up	−12.51	37.62	149.04	−10.36	14.40	60.66	−10.80	15.50	62.23
Roll	−0.027	0.794	1.926	0.268	2.115	5.540	0.019	0.752	1.667
Pitch	0.105	0.734	1.787	0.094	1.888	5.390	0.098	0.755	1.625
Yaw	0.732	0.891	2.213	−0.497	1.079	3.780	0.725	0.922	2.297

**Table 7:** D-42—GNSS-assisted—position (m) and attitude (deg) error statistics.

	INS			LiDAR			INS/LiDAR		
	Mean	RMSE	Max	Mean	RMSE	Max	Mean	RMSE	Max
East	−20.83	33.76	92.36	1.01	4.27	8.43	0.99	4.30	8.29
North	−3.93	25.72	79.29	−6.41	12.47	32.36	−6.41	12.47	32.31
Horizontal	28.63	42.44	121.73	8.97	13.18	33.44	8.97	13.19	33.35
Up	−3.42	6.49	18.70	−29.74	39.60	113.50	−3.40	6.48	18.60
Roll	−0.102	0.340	0.878	−0.340	2.290	6.046	−0.102	0.340	0.878
Pitch	−0.217	0.410	0.817	−3.056	4.817	10.521	−0.217	0.410	0.818
Yaw	0.139	0.313	0.822	0.043	0.799	1.891	0.139	0.313	0.821

**Table 8:** D-42—GNSS-assisted—position (m) and attitude (deg) error statistics.

	Proposed System		A-LOAM		LeGO-LOAM		F-LOAM	
	Mean	RMSE	Mean	RMSE	Mean	RMSE	Mean	RMSE
	D-28							
Horizontal	12.09	15.06	26.81	34.77	15.78	17.84	27.46	31.27
Up	10.61	13.57	10.94	13.34	16.72	18.92	7.99	11.00
	D-42							
Horizontal	27.86	32.15	167.42	201.33	575.94	715.11	67.55	87.35
Up	19.15	23.58	79.76	104.23	55.66	70.25	25.29	37.36
	D-101							
Horizontal	13.08	14.82	16.05	22.27	24.19	32.52	7.66	9.09
Up	4.86	6.03	19.59	28.57	19.13	29.33	5.18	7.82

## Data Availability

The data used in this study can be found at: http://www.cvlibs.net/datasets/kitti/ (accessed on 27 April 2022).

## Appendix A

### Appendix A.1. Residential Datasets

**Table A1:** D-18—position (m) and attitude (deg) error statistics.

	INS			LiDAR			INS/LiDAR		
	Mean	RMSE	Max	Mean	RMSE	Max	Mean	RMSE	Max
East	870.78	1252.61	3118.31	−0.50	3.34	7.35	−0.50	3.34	7.35
North	130.45	229.34	482.44	−9.51	11.88	20.71	−9.51	11.88	20.71
Horizontal	901.05	1273.43	3155.41	10.24	12.34	20.72	10.24	12.34	20.72
Up	17.78	23.83	56.71	3.71	4.20	10.54	3.94	4.72	9.81
Roll	0.119	0.740	1.763	0.274	1.151	2.482	0.130	0.735	1.767
Pitch	−0.036	0.641	1.651	−0.285	0.898	2.841	−0.039	0.631	1.639
Yaw	2.123	3.100	4.776	0.989	1.277	2.742	2.164	3.190	5.249

**Table A2:** D-19—position (m) and attitude (deg) error statistics.

	INS			LiDAR			INS/LiDAR		
	Mean	RMSE	Max	Mean	RMSE	Max	Mean	RMSE	Max
East	−3.71	4.39	7.75	2.75	2.83	4.10	2.74	2.83	4.10
North	0.29	1.73	5.01	−2.97	3.04	3.56	−2.97	3.04	3.56
Horizontal	4.00	4.71	9.23	4.08	4.15	5.27	4.08	4.15	5.27
Up	2.25	2.86	5.53	6.17	7.32	11.08	5.56	7.31	12.70
Roll	−0.047	0.120	0.311	−1.014	1.290	2.523	−0.050	0.122	0.312
Pitch	0.050	0.140	0.316	−1.218	1.409	2.753	0.047	0.136	0.309
Yaw	0.169	0.220	0.497	−0.575	0.638	1.456	0.170	0.221	0.497

**Table A3:** D-20—position (m) and attitude (deg) error statistics.

	INS			LiDAR			INS/LiDAR		
	Mean	RMSE	Max	Mean	RMSE	Max	Mean	RMSE	Max
East	−162.07	220.09	480.21	−0.90	1.00	1.95	−0.90	1.00	1.95
North	−283.97	375.19	827.29	1.68	1.86	3.80	1.68	1.86	3.80
Horizontal	327.41	434.98	956.56	2.02	2.11	4.01	2.02	2.11	4.01
Up	3.67	5.28	13.48	3.08	3.43	6.38	3.09	3.40	6.04
Roll	−0.066	0.592	0.921	0.444	1.218	1.980	−0.065	0.592	0.926
Pitch	−0.020	0.481	0.875	−0.292	0.953	1.936	−0.021	0.483	0.872
Yaw	1.439	1.448	1.708	−0.669	1.067	3.623	1.433	1.442	1.688

**Table A4:** D-22—position (m) and attitude (deg) error statistics.

	INS			LiDAR			INS/LiDAR		
	Mean	RMSE	Max	Mean	RMSE	Max	Mean	RMSE	Max
East	17.14	24.39	50.71	0.27	1.98	2.90	0.27	1.98	2.90
North	−76.01	107.52	267.92	−3.53	3.83	5.57	−3.53	3.83	5.57
Horizontal	78.00	110.25	272.67	4.03	4.31	6.11	4.03	4.31	6.11
Up	5.67	6.97	13.17	1.92	2.20	4.01	2.21	2.76	5.45
Roll	0.127	0.461	0.902	−0.313	0.592	1.373	0.126	0.452	0.888
Pitch	0.157	0.377	0.883	−0.304	0.614	1.479	0.153	0.370	0.864
Yaw	0.180	0.351	0.683	−0.016	0.733	1.734	0.174	0.346	0.677

**Table A5:** D-23—position (m) and attitude (deg) error statistics.

	INS			LiDAR			INS/LiDAR		
	Mean	RMSE	Max	Mean	RMSE	Max	Mean	RMSE	Max
East	15.55	21.10	47.48	0.45	0.63	1.30	0.45	0.63	1.30
North	6.63	8.25	13.72	0.83	1.29	2.20	0.83	1.29	2.20
Horizontal	18.00	22.65	47.68	1.20	1.43	2.26	1.20	1.43	2.26
Up	1.79	2.61	6.21	4.48	6.31	14.15	3.93	5.80	13.41
Roll	−0.193	0.336	0.619	−0.061	0.164	0.543	−0.200	0.346	0.646
Pitch	0.250	0.294	0.466	−1.471	1.726	3.227	0.240	0.284	0.444
Yaw	0.107	0.196	0.353	−1.015	1.027	1.380	0.108	0.198	0.363

**Table A6:** D-27_d—position (m) and attitude (deg) error statistics.

	INS			LiDAR			INS/LiDAR		
	Mean	RMSE	Max	Mean	RMSE	Max	Mean	RMSE	Max
East	47,110.14	62751.46	140945.00	1.98	20.16	54.11	1.98	20.16	54.11
North	13,282.11	15,974.94	23,896.15	2.13	23.92	62.92	2.13	23.92	62.92
Horizontal	49,127.97	64,752.95	142,305.11	24.38	31.28	74.57	24.38	31.29	74.56
Up	2234.59	3017.39	6928.42	7.24	8.23	17.69	22.01	23.81	45.11
Roll	0.120	6.341	10.456	0.296	1.775	6.077	0.143	5.749	10.012
Pitch	0.018	6.213	10.939	−0.196	1.399	4.327	−0.061	5.797	10.928
Yaw	−1.059	3.438	8.489	8.942	42.785	269.993	1.605	4.211	9.148

**Table A7:** D-33—position (m) and attitude (deg) error statistics.

	INS			LiDAR			INS/LiDAR		
	Mean	RMSE	Max	Mean	RMSE	Max	Mean	RMSE	Max
East	39.11	73.59	168.64	−0.18	5.07	8.62	−0.19	5.07	8.61
North	249.35	382.20	991.30	−0.96	6.63	13.08	−0.96	6.63	13.07
Horizontal	254.16	389.22	1005.54	7.92	8.35	13.38	7.92	8.35	13.37
Up	11.86	13.61	20.29	6.45	7.91	16.35	6.96	9.08	18.21
Roll	−0.030	0.435	1.142	−0.947	2.132	5.235	0.006	0.444	0.977
Pitch	−0.028	0.615	1.195	0.074	1.937	7.026	0.014	0.573	1.117
Yaw	−0.104	0.214	0.470	1.561	7.476	26.579	−0.124	0.270	0.702

**Table A8:** D-34—position (m) and attitude (deg) error statistics.

	INS			LiDAR			INS/LiDAR		
	Mean	RMSE	Max	Mean	RMSE	Max	Mean	RMSE	Max
East	−79.24	122.03	320.11	69.19	78.54	119.98	69.19	78.54	119.97
North	96.27	147.53	421.25	−4.50	9.12	17.64	−4.50	9.12	17.64
Horizontal	125.03	191.46	529.08	70.12	79.06	119.98	70.12	79.06	119.98
Up	9.30	13.07	23.81	9.81	11.40	21.19	9.65	11.75	21.31
Roll	0.223	0.367	1.146	−0.544	1.351	4.481	0.277	0.444	1.334
Pitch	−0.196	0.714	1.442	−0.466	2.728	4.549	−0.155	0.680	1.528
Yaw	0.068	0.169	0.425	10.368	10.388	12.454	−0.041	0.238	0.546

**Table A9:** D-34_d—position (m) and attitude (deg) error statistics.

	INS			LiDAR			INS/LiDAR		
	Mean	RMSE	Max	Mean	RMSE	Max	Mean	RMSE	Max
East	484.13	795.01	1556.31	−2.90	11.08	35.52	−2.90	11.08	35.52
North	743.81	1806.97	5738.22	−23.26	28.30	56.44	−23.26	28.30	56.44
Horizontal	1507.41	1974.13	5805.37	25.99	30.39	59.88	25.99	30.39	59.88
Up	165.96	316.38	1063.35	28.37	32.61	62.30	31.56	37.90	78.94
Roll	0.247	2.021	6.144	−0.089	3.475	11.262	0.077	2.430	8.920
Pitch	−0.070	2.644	6.298	−0.573	3.763	10.065	−0.040	3.570	9.032
Yaw	0.928	1.068	4.015	−2.987	12.188	66.465	0.423	1.337	3.942

**Table A10:** D-36—position (m) and attitude (deg) error statistics.

	INS			LiDAR			INS/LiDAR		
	Mean	RMSE	Max	Mean	RMSE	Max	Mean	RMSE	Max
East	15.94	21.86	50.58	−0.85	1.28	2.45	−0.85	1.28	2.45
North	−67.85	100.41	252.75	1.71	2.11	4.20	1.71	2.11	4.20
Horizontal	69.90	102.77	257.77	2.28	2.46	4.21	2.28	2.46	4.21
Up	4.60	5.94	10.51	1.09	1.28	2.54	0.88	1.23	3.04
Roll	−0.387	0.464	0.743	−0.987	1.127	1.929	−0.400	0.480	0.767
Pitch	0.198	0.507	0.795	−0.580	0.916	1.892	0.186	0.494	0.770
Yaw	0.194	0.220	0.444	0.024	0.386	1.050	0.193	0.217	0.446

**Table A11:** D-39—position (m) and attitude (deg) error statistics.

	INS			LiDAR			INS/LiDAR		
	Mean	RMSE	Max	Mean	RMSE	Max	Mean	RMSE	Max
East	−8.03	10.37	21.34	−6.28	7.47	12.20	−6.28	7.47	12.20
North	11.53	17.57	44.76	5.28	6.28	10.13	5.28	6.28	10.13
Horizontal	14.41	20.40	49.59	8.29	9.76	15.85	8.29	9.76	15.85
Up	6.15	7.61	14.47	1.69	2.11	4.17	3.24	3.60	4.73
Roll	−0.361	0.392	0.615	1.045	1.337	2.870	−0.360	0.391	0.614
Pitch	−0.254	0.306	0.493	−0.998	1.175	2.153	−0.245	0.295	0.491
Yaw	−0.077	0.106	0.310	2.898	2.937	3.481	−0.077	0.107	0.310

**Table A12:** D-46—position (m) and attitude (deg) error statistics.

	INS			LiDAR			INS/LiDAR		
	Mean	RMSE	Max	Mean	RMSE	Max	Mean	RMSE	Max
East	−0.77	0.99	2.14	−0.26	0.44	0.88	−0.26	0.44	0.88
North	0.32	0.42	1.11	−0.30	0.39	0.69	−0.30	0.39	0.69
Horizontal	0.84	1.08	2.41	0.57	0.59	0.91	0.56	0.58	0.91
Up	0.39	0.48	1.00	1.34	1.35	1.62	0.34	0.41	0.78
Roll	0.037	0.068	0.186	−0.182	0.441	1.061	0.037	0.068	0.186
Pitch	−0.034	0.062	0.104	0.074	0.298	0.614	−0.034	0.062	0.103
Yaw	−0.107	0.153	0.316	−0.776	0.990	1.858	−0.108	0.154	0.316

**Table A13:** D-61—position (m) and attitude (deg) error statistics.

	INS			LiDAR			INS/LiDAR		
	Mean	RMSE	Max	Mean	RMSE	Max	Mean	RMSE	Max
East	−125.23	212.27	618.43	0.26	0.50	1.35	0.26	0.50	1.35
North	9.36	18.80	57.37	1.63	1.90	3.07	1.63	1.90	3.07
Horizontal	126.06	213.10	621.09	1.70	1.97	3.27	1.70	1.97	3.27
Up	11.66	19.69	55.26	3.79	4.60	10.40	5.91	8.07	16.39
Roll	−0.603	1.168	2.447	−0.373	0.497	1.634	−0.387	0.873	1.930
Pitch	2.201	2.828	4.468	−1.376	1.750	3.034	2.258	2.905	4.519
Yaw	0.227	0.328	0.676	−0.271	0.502	1.441	0.227	0.331	0.676

**Table A14:** D-64—position (m) and attitude (deg) error statistics.

	INS			LiDAR			INS/LiDAR		
	Mean	RMSE	Max	Mean	RMSE	Max	Mean	RMSE	Max
East	−4.70	5.72	13.46	−7.50	9.62	19.22	−7.50	9.62	19.22
North	−26.14	38.60	94.03	8.64	10.43	16.53	8.64	10.43	16.53
Horizontal	26.77	39.02	94.99	11.66	14.19	24.06	11.66	14.19	24.06
Up	1.16	1.37	2.42	1.65	2.08	6.14	1.43	1.69	4.15
Roll	0.152	0.358	0.817	−1.510	2.038	3.508	0.160	0.352	0.815
Pitch	0.226	0.270	0.535	−1.667	2.143	4.440	0.214	0.256	0.503
Yaw	−0.021	0.101	0.306	3.245	3.288	4.696	−0.023	0.100	0.293

**Table A15:** D-79—position (m) and attitude (deg) error statistics.

	INS			LiDAR			INS/LiDAR		
	Mean	RMSE	Max	Mean	RMSE	Max	Mean	RMSE	Max
East	0.60	0.68	1.19	0.32	0.33	0.43	0.32	0.33	0.43
North	0.49	0.58	1.12	0.16	0.20	0.30	0.16	0.20	0.30
Horizontal	0.78	0.89	1.63	0.38	0.39	0.51	0.37	0.39	0.51
Up	0.12	0.16	0.31	1.90	1.92	2.15	0.14	0.17	0.32
Roll	−0.063	0.103	0.171	0.968	1.005	1.865	−0.063	0.103	0.172
Pitch	−0.044	0.071	0.118	−0.056	0.165	0.491	−0.044	0.071	0.118
Yaw	−0.005	0.051	0.113	−0.238	0.440	0.810	−0.005	0.051	0.113

**Table A16:** D-86—position (m) and attitude (deg) error statistics.

	INS			LiDAR			INS/LiDAR		
	Mean	RMSE	Max	Mean	RMSE	Max	Mean	RMSE	Max
East	23.17	32.12	66.08	3.23	4.02	8.01	3.23	4.02	8.01
North	−43.10	63.11	141.80	−1.75	2.02	3.18	−1.75	2.02	3.18
Horizontal	49.42	70.81	156.44	3.79	4.50	8.11	3.79	4.50	8.11
Up	7.05	9.22	19.31	0.45	0.53	0.94	1.30	1.45	2.42
Roll	−0.257	0.421	0.907	1.186	1.689	3.914	−0.253	0.425	0.913
Pitch	−0.125	0.211	0.385	0.209	1.514	4.972	−0.150	0.231	0.437
Yaw	0.685	0.717	1.623	1.351	1.432	2.613	0.682	0.713	1.604

**Table A17:** D-87—position (m) and attitude (deg) error statistics.

	INS			LiDAR			INS/LiDAR		
	Mean	RMSE	Max	Mean	RMSE	Max	Mean	RMSE	Max
East	14.96	30.21	93.41	−0.37	0.61	1.78	−0.37	0.61	1.78
North	−22.19	32.94	92.80	−0.78	1.16	2.63	−0.78	1.16	2.62
Horizontal	28.67	44.70	131.67	1.09	1.31	2.68	1.09	1.31	2.67
Up	4.56	6.68	16.36	1.30	1.57	3.47	1.99	2.42	4.32
Roll	0.491	0.672	1.449	−3.105	3.711	5.740	0.453	0.616	1.328
Pitch	0.094	0.283	0.843	−0.591	1.050	3.277	0.063	0.239	0.689
Yaw	−0.063	0.560	1.137	0.638	0.855	1.616	−0.061	0.557	1.122

### Appendix A.2. Highway Datasets

**Table A18:** D-04—position (m) and attitude (deg) error statistics.

	INS			LiDAR			INS/LiDAR		
	Mean	RMSE	Max	Mean	RMSE	Max	Mean	RMSE	Max
East	2.388	3.190	7.487	5.890	6.397	8.745	5.894	6.405	8.757
North	−5.394	7.385	16.745	−5.845	6.496	9.873	−5.849	6.507	9.895
Horizontal	5.908	8.045	18.343	8.317	9.117	13.184	8.320	9.130	13.209
Up	0.321	0.344	0.481	2.894	3.068	4.575	0.327	0.350	0.484
Roll	0.142	0.149	0.225	0.096	0.410	0.919	0.142	0.149	0.225
Pitch	0.065	0.069	0.120	−0.743	0.834	1.220	0.065	0.069	0.120
Yaw	0.076	0.099	0.169	−3.196	3.203	3.409	0.076	0.099	0.169

**Table A19:** D-15—position (m) and attitude (deg) error statistics.

	INS			LiDAR			INS/LiDAR		
	Mean	RMSE	Max	Mean	RMSE	Max	Mean	RMSE	Max
East	1.97	2.56	5.53	−0.94	0.95	1.29	−0.91	0.94	1.51
North	3.29	4.45	10.38	−0.86	0.88	1.31	−0.84	0.87	1.48
Horizontal	3.84	5.14	11.77	1.28	1.30	1.84	1.24	1.28	2.12
Up	0.48	0.60	1.30	3.53	3.94	7.99	0.48	0.60	1.31
Roll	0.008	0.027	0.065	1.165	1.169	1.394	0.008	0.027	0.065
Pitch	−0.184	0.203	0.400	−0.745	0.899	1.764	−0.184	0.203	0.400
Yaw	0.053	0.071	0.176	−0.372	0.392	0.635	0.053	0.071	0.176

**Table A20:** D-16—position (m) and attitude (deg) error statistics.

	INS			LiDAR			INS/LiDAR		
	Mean	RMSE	Max	Mean	RMSE	Max	Mean	RMSE	Max
East	−0.61	0.81	1.37	−1.34	1.61	2.86	−1.34	1.61	2.86
North	2.45	3.34	7.00	−1.08	1.13	1.69	−1.07	1.12	1.64
Horizontal	2.55	3.43	7.13	1.77	1.97	3.32	1.76	1.97	3.30
Up	1.58	1.90	3.33	2.89	4.14	9.92	1.58	1.90	3.33
Roll	−0.014	0.050	0.139	−1.496	1.811	3.116	−0.014	0.050	0.139
Pitch	−0.072	0.119	0.220	−1.261	1.492	2.709	−0.072	0.119	0.220
Yaw	0.025	0.042	0.111	0.187	0.203	0.384	0.025	0.042	0.111

**Table A21:** D-28—position (m) and attitude (deg) error statistics.

	INS			LiDAR			INS/LiDAR		
	Mean	RMSE	Max	Mean	RMSE	Max	Mean	RMSE	Max
East	−2.72	3.63	7.94	4.86	4.97	7.24	4.81	4.97	8.74
North	−6.63	9.31	23.20	−6.86	6.98	8.22	−6.84	7.00	8.06
Horizontal	7.17	10.00	24.52	8.54	8.57	9.19	8.50	8.58	10.05
Up	1.31	1.54	2.78	7.72	9.92	22.09	1.32	1.56	2.82
Roll	−0.135	0.146	0.238	−0.595	1.144	2.061	−0.135	0.146	0.238
Pitch	0.118	0.150	0.370	−1.883	2.207	3.835	0.118	0.150	0.370
Yaw	0.294	0.317	0.450	−0.032	0.516	1.059	0.294	0.317	0.450

**Table A22:** D-29—position (m) and attitude (deg) error statistics.

	INS			LiDAR			INS/LiDAR		
	Mean	RMSE	Max	Mean	RMSE	Max	Mean	RMSE	Max
East	−4.71	6.59	13.50	−0.70	0.83	1.36	−0.70	0.84	1.37
North	18.41	25.28	58.60	0.12	0.44	0.79	0.14	0.46	0.80
Horizontal	19.04	26.12	60.13	0.82	0.94	1.51	0.83	0.96	1.53
Up	3.28	3.73	5.54	4.43	4.61	5.85	3.28	3.73	5.52
Roll	0.184	0.322	0.658	0.610	0.687	1.245	0.184	0.322	0.658
Pitch	−0.158	0.281	0.378	−0.243	0.806	1.250	−0.158	0.281	0.378
Yaw	−0.084	0.307	1.660	−0.934	1.329	3.620	−0.084	0.306	1.660

**Table A23:** D-32—position (m) and attitude (deg) error statistics.

	INS			LiDAR			INS/LiDAR		
	Mean	RMSE	Max	Mean	RMSE	Max	Mean	RMSE	Max
East	2.31	2.87	5.45	0.73	0.77	1.06	0.74	0.77	1.05
North	1.18	1.39	2.36	0.41	0.85	2.18	0.41	0.84	2.13
Horizontal	2.63	3.19	5.75	1.04	1.15	2.25	1.04	1.14	2.20
Up	2.45	3.36	7.56	8.70	10.46	21.40	2.45	3.36	7.56
Roll	−0.019	0.072	0.196	0.760	0.767	1.042	−0.019	0.072	0.196
Pitch	−0.038	0.077	0.194	−1.411	1.634	2.996	−0.038	0.077	0.194
Yaw	0.023	0.050	0.093	−0.409	0.492	0.863	0.023	0.050	0.093
